# Structure-based design, synthesis, and evaluation of the biological activity of novel phosphoroorganic small molecule IAP antagonists

**DOI:** 10.1007/s10637-020-00923-4

**Published:** 2020-04-08

**Authors:** Agnieszka Łupicka-Słowik, Mateusz Psurski, Renata Grzywa, Monika Cuprych, Jarosław Ciekot, Waldemar Goldeman, Elżbieta Wojaczyńska, Jacek Wojaczyński, Józef Oleksyszyn, Marcin Sieńczyk

**Affiliations:** 1 Faculty of Chemistry, Department of Organic and Medicinal Chemistry, Wrocław University of Science and Technology, Wybrzeże Wyspiańskiego 27, 50-370 Wrocław, Poland; 2 Department of Experimental Oncology, Hirszfeld Institute of Immunology and Experimental Therapy, Polish Academy of Sciences, Weigla 12, 53-114 Wrocław, Poland; 3 Faculty of Chemistry, Department of Physical and Quantum Chemistry, Wrocław University of Science and Technology, Wybrzeże Wyspiańskiego 27, 50-370 Wrocław, Poland; 4 Department of Chemistry, University of Wrocław, F. Joliot-Curie 14, 50-383 Wrocław, Poland

**Keywords:** IAP antagonist, Smac mimetic, Apoptosis, Phosphoroorganic

## Abstract

One of the strategies employed by novel anticancer therapies is to put the process of apoptosis back on track by blocking the interaction between inhibitor of apoptosis proteins (IAPs) and caspases. The activity of caspases is modulated by the caspases themselves in a caspase/procaspase proteolytic cascade and by their interaction with IAPs. Caspases can be released from the inhibitory influence of IAPs by proapoptotic proteins such as secondary mitochondrial activator of caspases (Smac) that share an IAP binding motif (IBM). The main purpose of the present study was the design and synthesis of phosphorus-based peptidyl antagonists of IAPs that mimic the endogenous Smac protein, which blocks the interaction between IAPs and caspases. Based on the structure of the IAP antagonist and recently reported thiadiazole derivatives, we designed and evaluated the biochemical properties of a series of phosphonic peptides bearing the *N*-Me-Ala-Val/Chg-Pro-OH motif (Chg: cyclohexylglycine). The ability of the obtained compounds to interact with the binding groove of the X-linked inhibitor of apoptosis protein baculovirus inhibitor of apoptosis protein repeat (XIAP BIR3) domain was examined by a fluorescence polarization assay, while their potential to induce autoubiquitination followed by proteasomal degradation of cellular IAP1 was examined using the MDA-MB-231 breast cancer cell line. The highest potency against BIR3 was observed among peptides containing C-terminal phosphonic phenylalanine analogs, which displayed nanomolar K_i_ values. Their antiproliferative potential as well as their proapoptotic action, manifested by an increase in caspase-3 activity, was examined using various cell lines.

**Electronic supplementary material:**

The online version of this article (10.1007/s10637-020-00923-4) contains supplementary material, which is available to authorized users.

## Introduction

Apoptosis, a process of programmed cell death, is a fundamental mechanism under precise homeostatic control. It assures a delicate balance between cell survival and death, which is crucial for embryogenesis as well as for sustaining a constant number of mature cells while preventing the spread of infectious diseases. Suppression of apoptosis is utilized by cancer cells for their uncontrolled proliferation and is observed during the development of autoimmune diseases [1, 2]. Conversely, excessive apoptosis may be linked to various neurodegenerative disorders and diabetes [3, 4].

There are two pathways that lead to the activation of apoptosis. The extrinsic pathway is initiated through the activation of death receptors that belong to the TNF receptor superfamily, such as tumor necrosis factor (TNF)-related apoptosis-inducing ligand (TRAIL) or Fas. The signal is then transmitted *via* Fas-associated death domain protein (FADD)-dependent activation of caspase-8 and caspase-10, which in turn proteolytically process executioner caspase-3 and caspase-7, leading to apoptosis [5–8]. The intrinsic (mitochondrial) pathway is activated by cell stress, such as DNA damage, cytoskeletal disruption, accumulation of unfolded proteins, hypoxia or metabolic stress, which results in permeabilization of the outer mitochondrial membrane [9–11]. As a consequence, mitochondrial intermembrane cytochrome c and secondary mitochondrial activator of caspases (Smac) proteins are released into the cytosol. Cytochrome c is involved in the formation of the apoptosome followed by the activation of caspase-9 and, finally, caspase-3 and caspase-7, whereas the Smac protein binds to the X-linked inhibitor of apoptosis protein (XIAP), which liberates the caspases from inhibitor control [8].

XIAP is a member of the family of inhibitor of apoptosis proteins (IAPs) that are considered to be negative regulators of caspases and cell death [12]. Among the human IAPs, eight different proteins have been distinguished: neuronal apoptosis inhibitory protein (NAIP/BIRC1), cellular IAP1 (cIAP1/BIRC2), cellular IAP2 (cIAP2/BIRC3), survivin (BIRC5), BIR-containing ubiquitin-conjugating enzyme (BRUCE/Apollon/BIRC6), melanoma IAP (ML-IAP/BIRC7), IAP-like protein 2 (ILP2/BIRC8) and X-chromosome-linked IAP (XIAP/BIRC4) [13]. The structures of IAPs are characterized by the presence of at least one zinc-binding baculoviral domain (baculovirus inhibitor of apoptosis protein repeat, BIR), which is essential for their antiapoptotic activity [14, 15]. Additionally, some IAPs contain a really interesting new gene (RING) finger domain that promotes ubiquitination of IAPs and other associated proteins, a ubiquitin (Ub)-associated domain (UBA) capable of binding the poly-Ub chains and a conserved caspase recruitment domain (CARD) [16]. The structural organization of human IAP proteins is the subject of several excellent reviews [17–21].

**Fig. 1:**
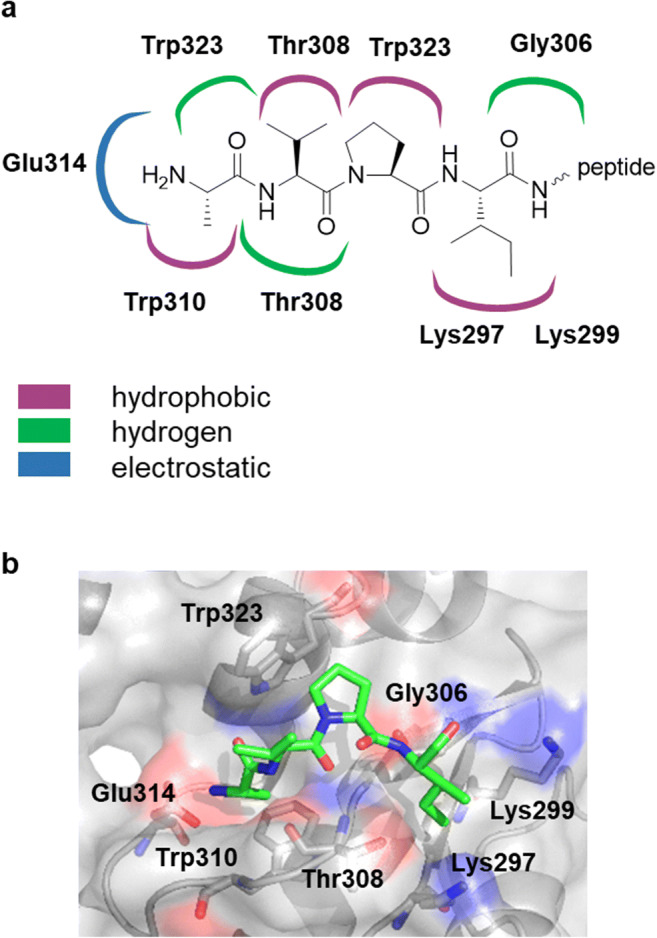
The interaction network between the *N*-terminal tetrapeptide (Ala-Val-Pro-Ile) of Smac and the XIAP BIR3 domain (based on the NMR data) [30] (**a**) and representation of the peptide-binding groove of the BIR3 domain containing the bound N-terminal AVPI motif of Smac (based on 1G73.pdb) (**b**) [31]

Increased expression of XIAP has been observed during the neoplastic processes of prostate cancer [22], non-small-cell lung carcinoma [23, 24], acute myeloid leukemia [25] and acute mixed lineage leukemia [26]. In addition, the XIAP expression level correlates with tissue resistance to chemotherapeutic agents [27]. The XIAP protein contains three different BIR domains (BIR1-BIR3), a C-terminal RING domain and a UBA domain. The interdomain fragments of BIR1 and BIR2 together with the BIR2 domain act as an inhibitor of caspase-3 and caspase-7, whereas the BIR3 domain is able to inhibit caspase-9, preventing the dimerization of caspase-9 [28, 29]. The inhibitory activity of the BIR3 domain can be diminished by Smac released into the cytosol. The key role in molecular recognition between the XIAP BIR3 domain and Smac is played by the *N*-terminal fragment (AVPI) of the Smac protein [30]. The fundamentals of these complex interactions have been determined by crystal structure analysis [31] and NMR spectroscopy [30] (Fig. 1).

Several potential therapeutics and therapy-supportive agents acting as monovalent or bivalent Smac mimetics are currently being tested in clinical trials for cancer treatment [32, 33]. Monovalent compounds mimic the binding of a single AVPI motif to BIR domains, whereas bivalent mimetics are composed of two such binding units. Although bivalent antagonists are more potent than monovalent compounds, their pharmacological profile, including bioavailability, is less favorable [34].

**Fig. 2:**
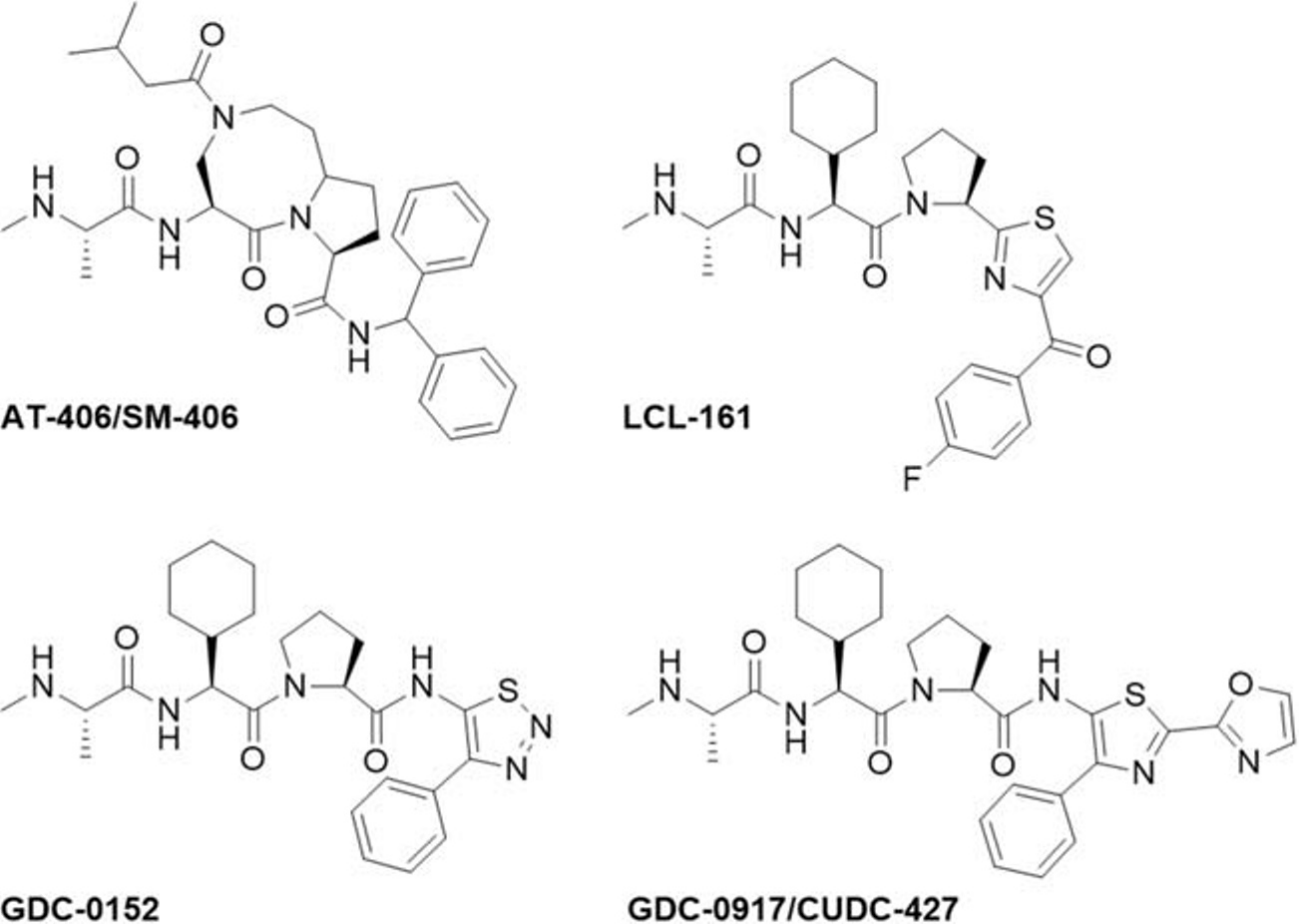
Chemical structures of previously reported monovalent Smac mimetics with therapeutic potential: AT-406/SM-406 [35], LCL-161 [36, 37], GDC-0152 [38, 39], and GDC-0917/CUDC-427 [40, 41]

Work in our laboratory has focused on the design and synthesis of monovalent phosphoroorganic-based Smac analogs as antagonists of the XIAP protein. These analogs mimic the interaction of the endogenous AVPI binding motif of Smac with the BIR3 domain. Our attempts to introduce phosphoroorganic function into the IAP antagonist scaffold were based on previously reported structures (Fig. 2) [35–41].

Herein, we report the design, synthesis and biological activity of novel peptidyl derivatives of phosphine oxides and α-aminoalkylphosphonic acid esters as antagonists of the XIAP BIR3 domain. The ability of these compounds to interact with the binding groove of the XIAP BIR3 domain was evaluated *via* a fluorescence polarization assay. Based on the fluorescence polarization data, we selected the synthesized phosphoroorganic derivative compounds that displayed the most favorable kinetic parameters and further examined their ability to induce cellular cIAP1 autoubiquitylation and proteasomal degradation. Additionally, we ascertained their antiproliferative activity as well as their proapoptotic potential in a chemoresistant, highly aggressive breast cancer cell line.

## Results and discussion

**Fig. 3:**
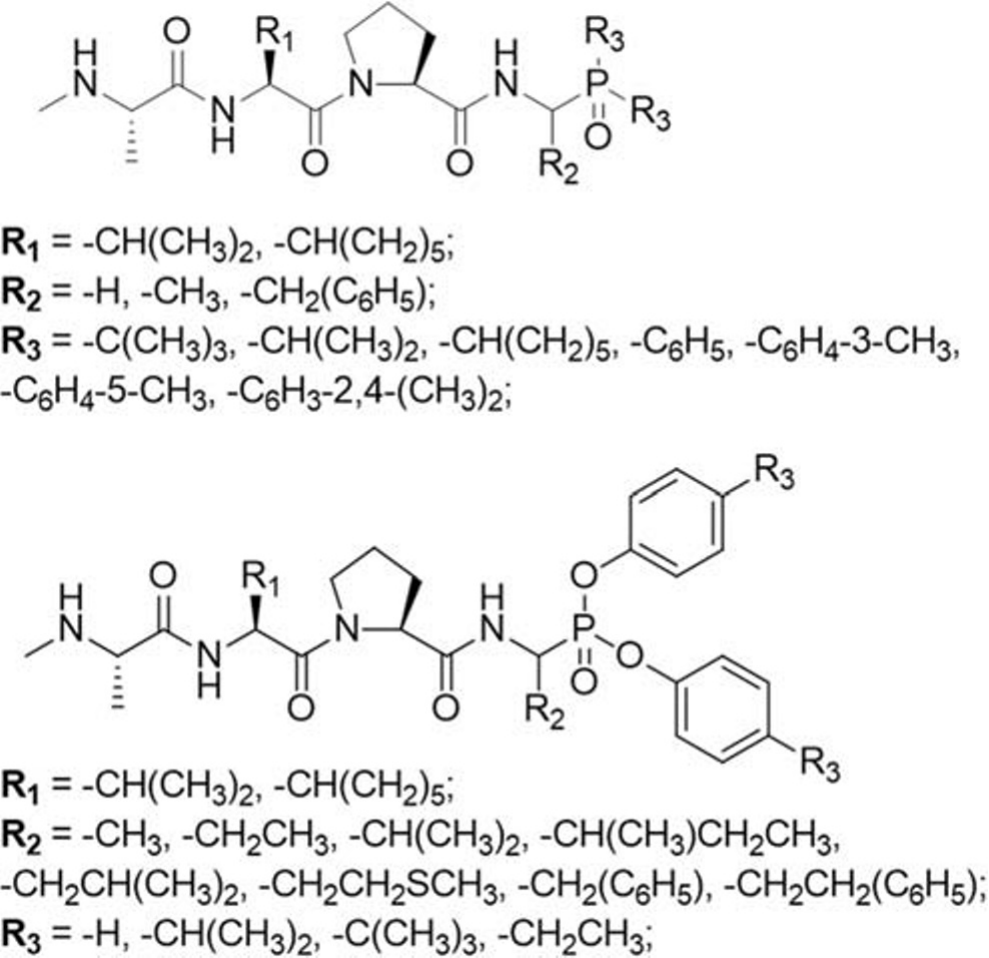
General structure of phosphoroorganic peptide derivatives designed as potential IAP antagonists

Based upon the structure of the endogenous IAP antagonist Smac and the recently discovered thiadiazole derivatives GDC-0152 [38] and LCL-161 [42], we introduced various C-terminal phosphoroorganic functionalities into *N*-Me-Ala-Val/Chg-Pro-OH scaffolds (Chg: cyclohexylglycine). As shown in previous studies, the methyl group of the Ala residue fits into the hydrophobic pocket formed by the Leu131, Trp134 and Glu143 side chains of ML-IAP, and because of steric limitations, it is difficult to replace with more complex substituents [38]. The amino group of Ala interacts with the carboxylates of Asp138 and Glu143 in ML-IAP, Glu314 in XIAP and Asp320 and Glu325 in cIAP1 [38, 43, 44]. *N*-methylation of the *N*-terminal Ala leads to increased cellular stability of these peptidyl compounds without reducing affinity for the BIR domain [45] and is one of the strategies used for improving pharmacokinetic parameters of potential intercalators [46]. The proline residue assures the most favorable orientation of Ala; however, the Chg-Pro dipeptide tolerates some level of structural variation. The Chg/Val side chain has a bulky character, and the rotation of this residue is restricted by Pro. According to Flygare et al., there is little difference in the binding affinity between corresponding compounds with endogenous Val or Chg derivatives, although compounds with a larger side chain at this position might exhibit drug-like properties because of the improved proteolytic stability of the peptide bond between Chg and Pro [38]. Contained within the XIAP BIR3 domain is a hydrophobic pocket between the aliphatic region of the Lys297 and Lys299 side chains and Leu292, Gly306 and Thr308 that enables interactions with aromatic rings. This area is responsible for interactions with the Ile side chain in the Ala-Val-Pro-Ile sequence of Smac and is the region in which we decided to introduce a phosphoroorganic moiety. The data obtained by Flygare et al. revealed that the presence of an aromatic ring at this position rather than on the side chain of the endogenously occurring isoleucine is preferred [38]. The presented data indicated that a phosphoroorganic, bulky moiety introduced to the *N*-Me-Ala-Val/Chg-Pro-OH scaffold can be an alternative to the endogenous Ile residue. The designed and synthesized structures of aliphatic and aromatic phosphine oxide peptidomimetics, as well as phosphonate derivatives, are schematically shown in Fig. 3.

The first step of the present study was the preparation of the peptidyl scaffolds (*N*-Me-Ala-Val-Pro-OH (**1**) and *N*-Me-Ala-Chg-Pro-OH (**2**)) that would then be used for further synthesis of the antagonists; the tetrapeptide H_2_N-Ala-Val-Pro-Trp-OH (**3**), which was used as a reference compound for the fluorescence polarization competition assay; and the fluorescent probe H_2_N-Ala-Val-Pro-Dpm-Ala-Lys(5(6)-Fam)-Lys-NH_2_ (**4**). All peptides were synthesized manually on a solid support. The *N*-terminal methyl group was introduced into the H_2_N-Ala-Val/Chg-Pro-OH peptides *via* the Mitsunobu reaction [47] with *o*-nitrobenzenesulfonyl as the protecting group and MeOH as the methyl donor yielding *N*-Me-Ala-Val-Pro-OH (**1**) and *N*-Me-Ala-Chg-Pro-OH (**2**), which were further modified with various phosphoroorganic moieties. Modification of the peptide probe with fluorescein was achieved through selective removal of the 4-methyltrityl protecting group (Mtt) [48] followed by fluorescein conjugation to the ε-amino group of Lys, yielding the peptide H_2_N-Ala-Val-Pro-Dpm-Ala-Lys(5(6)-Fam)-Lys-NH_2_ (**4**) required for fluorescence polarization assays.

Different phosphoroorganic derivatives were designed and synthesized for introduction as the C-terminal functionality of the *N*-methylated peptide scaffolds. The α-aminoalkylphosphine oxides were obtained from tritylimines and phosphine oxides followed by a trityl group deprotection, while the diaryl esters of α-aminoalkylphosphonic acids were synthesized *via* the α-amidoalkylation reaction described by Oleksyszyn [49].

**Fig. 4:**
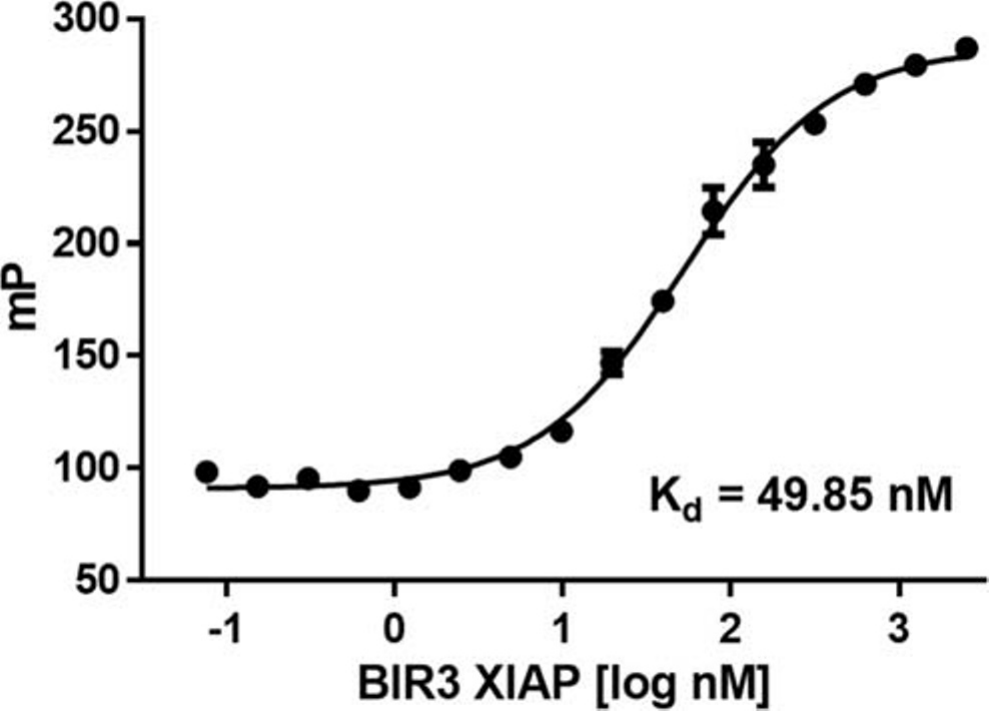
Determination of the binding affinity of fluorescent probe **4** and the XIAP BIR3 domain. The assay was performed at a constant fluorescent probe concentration (2 nM) and serially diluted XIAP BIR3 domain (ranging from 2.5 µM to 0.0763 nM)

A fluorescence polarization assay was used to examine the ability of the obtained compounds to interact with the binding groove located on the surface of the XIAP BIR3 domain. First, the conditions of the assay were optimized, and the K_d_ value for the protein-probe interaction was determined. The binding assay was performed with serial dilutions of the XIAP BIR3 domain (2.5 µM to 0.0763 nM) and fixed concentrations of fluorescent probe **4** (2 nM). We determined the affinity of the fluorescent probe and the XIAP BIR3 domain (K_d_ = 49.85 ± 5.37 nM) with a dynamic range of ΔmP = 196.2 ± 4.5 (Fig. 4).

We also analyzed the interaction of the synthesized phosphoroorganic peptide derivatives with the binding groove of the BIR3 domain. As reference compounds, commercially available XIAP antagonists GDC-0152 and LCL-161 were used together with tetrapeptide H_2_N-Ala-Val-Pro-Trp-OH (**3**), which is characterized by its high affinity to BIR domains of IAP family proteins [50], and control tripeptides **1** and **2**, which did not interact with the XIAP BIR3 domain at a significant level. The selection of H_2_N-Ala-Val-Pro-Trp-OH as a scaffold peptide instead of H_2_N-Ala-Val-Pro-Ile-OH (which endogenously binds to the BIR domain as the Smac *N*-terminal part) was dictated by its higher binding affinity to the BIR domain despite its low biological activity in cell-based assays [50, 51]. The kinetic parameters (EC_50_ and K_i_) of the obtained phosphoroorganic derivatives were determined by a fluorescence polarization assay performed at constant concentrations of the XIAP BIR3 domain (50 nM) and fluorescent probe **4** (2 nM), while the concentrations of potential antagonists ranged from 50 µM to 0.640 nM (for screening purposes) or from 50 µM to 0.0537 nM (for detailed analysis). The fluorescence polarization results were used for the determination of the Z´ factor, which provides information regarding the dynamic range and data quality (Z´ = 0.737; Supplementary Figure S1, Supplementary Materials). Most of the phosphoroorganic compounds exhibited the ability to interact with a binding groove on the surface of the XIAP BIR3 domain at a concentration of 50 µM, as manifested by the inhibition of the protein-probe interaction. Although the obtained data make it difficult to distinguish which R_1_ substituent structure (isopropyl or cyclohexyl, Fig. 3) has the greatest impact on the organophosphorus derivative affinity toward the target protein, slightly higher activity was observed for compounds containing a cyclic R_1_ ring (Tables 1 and 2). Exceptions may include compounds **41** and **42**, which were found to be the most active derivatives obtained in this study. One possible explanation for the increased activity of these two compounds could be that the phosphonic phenylalanine can influence the ability of the N-terminal residue of the peptide to interact with the binding groove of the BIR domain.

**Table 1:** Kinetic parameters of α-aminoalkylphosphine oxide peptidyl derivatives

No.	Structure			log EC_50_ [µM]	EC_50_ [µM]	K_i_ [µM]	% Inh (50 µM)

	**R**_**1**_	**R**_**2**_	**R**_**3**_				
**5**	-CH(CH_3_)_2_	-H	-C(CH_3_)_3_	1.471 ± 0.043	29.58	14.68	63.2
**6**	-CH(CH_2_)_5_	-H	-C(CH_3_)_3_	1.299 ± 0.044	19.90	9.87	72.0
**7**	-CH(CH_3_)_2_	-H	-CH(CH_3_)_2_	-	-	-	32.0
**8**	-CH(CH_2_)_5_	-H	-CH(CH_3_)_2_	-	-	-	41.3
**9**	-CH(CH_3_)_2_	-H	-CH(CH_2_)_5_	-	-	-	25.9
**10**	-CH(CH_2_)_5_	-H	-CH(CH_2_)_5_	-	-	-	24.2
**11**	-CH(CH_3_)_2_	-H	-C_6_H_5_	1.468 ± 0.055	29.33	14.55	63.6
**12**	-CH(CH_2_)_5_	-H	-C_6_H_5_	1.420 ± 0.051	26.29	13.04	67.1
**13**	-**CH(CH**_**3**_**)**_**2**_	-**CH**_**2**_**(C**_**6**_**H**_**5**_**)**	-**C**_**6**_**H**_**5**_	**0.186 ± 0.080**	**1.53**	**0.75**	**100.0**
**14**	-**CH(CH**_**2**_**)**_**5**_	-**CH**_**2**_**(C**_**6**_**H**_**5**_**)**	-**C**_**6**_**H**_**5**_	**0.118 ± 0.059**	**1.31**	**0.64**	**100.0**
**15**	-CH(CH_3_)_2_	-H	-C_6_H_4_-4-CH_3_	1.489 ± 0.045	30.83	15.30	61.6
**16**	-CH(CH_2_)_5_	-H	-C_6_H_4_-4-CH_3_	1.606 ± 0.045	40.32	20.01	57.0
**17**	-CH(CH_2_)_5_	-CH_3_	-C_6_H_4_-4-CH_3_	1.285 ± 0.041	19.25	9.55	74.0
**18**	-CH(CH_3_)_2_	-H	-C_6_H_4_-2-CH_3_	1.466 ± 0.042	29.27	14.52	62.0
**19**	-CH(CH_2_)_5_	-H	-C_6_H_4_-2-CH_3_	1.448 ± 0.044	28.03	13.91	66.2
**20**	-CH(CH_3_)_2_	-H	-C_6_H_3_-3,5-(CH_3_)_2_	1.681 ± 0.042	47.96	23.80	50.0
**21**	-CH(CH_2_)_5_	-H	-C_6_H_3_-3,5-(CH_3_)_2_	1.353 ± 0.032	22.54	11.18	74.4
**22**	-CH(CH_3_)_2_	-CH_3_	-C_6_H_3_-3,5-(CH_3_)_2_	1.511 ± 0.051	32.46	16.11	59.2

**Table 2:** Kinetic parameters of α-aminoalkylphosphonate diaryl ester peptidyl derivatives

No.	Structure			log EC_50_ [µM]	EC_50_ [µM]	K_i_ [µM]	% Inh (50 µM)

	**R**_**1**_	**R**_**2**_	**R**_**3**_				
**23**	-CH(CH_3_)_2_	-CH_3_	-CH_2_CH_3_	1.535 ± 0.031	34.25	17.00	64.1
**24**	-CH(CH_2_)_5_	-CH_3_	-CH_2_CH_3_	1.313 ± 0.053	20.54	10.19	74.1
**25**	-CH(CH_3_)_2_	-CH_3_	-CH(CH_3_)_2_	1.342 ± 0.026	21.98	10.90	81.8
**26**	-CH(CH_2_)_5_	-CH_3_	-CH(CH_3_)_2_	1.297 ± 0.033	19.83	9.84	85.8
**27**	-CH(CH_3_)_2_	-CH_3_	-C(CH_3_)_3_	1.328 ± 0.039	21.26	10.54	83.3
**28**	-CH(CH_2_)_5_	-CH_3_	-C(CH_3_)_3_	1.427 ± 0.041	26.76	13.27	77.3
**29**	-CH(CH_3_)_2_	-CH_3_	-H	1.503 ± 0.042	31.86	15.81	62.1
**30**	-CH(CH_2_)_5_	-CH_3_	-H	1.481 ± 0.060	30.24	15.00	69.0
**31**	-CH(CH_3_)_2_	-CH_2_CH_3_	-H	1.580 ± 0.056	37.97	18.84	57.0
**32**	-CH(CH_2_)_5_	-CH_2_CH_3_	-H	1.380 ± 0.074	23.97	11.89	71.0
**33**	-CH(CH_3_)_2_	-CH(CH_3_)_2_	-H	1.543 ± 0.052	34.96	17.35	58.5
**34**	-CH(CH_2_)_5_	-CH(CH_3_)_2_	-H	1.378 ± 0.034	23.85	11.83	70.5
**35**	-CH(CH_3_)_2_	-CH(CH_3_)CH_2_CH_3_	-H	1.251 ± 0.037	17.84	8.84	77.4
**36**	-CH(CH_2_)_5_	-CH(CH_3_)CH_2_CH_3_	-H	1.211 ± 0.036	16.24	8.05	81.4
**37**	-CH(CH_3_)_2_	-CH_2_CH(CH_3_)_2_	-H	1.449 ± 0.062	28.13	13.96	67.4
**38**	-CH(CH_2_)_5_	-CH_2_CH(CH_3_)_2_	-H	1.026 ± 0.051	10.61	5.25	84.2
**39**	-CH(CH_3_)_2_	-CH_2_CH_2_SCH_3_	-H	1.418 ± 0.041	26.20	13.00	65.8
**40**	-CH(CH_2_)_5_	-CH_2_CH_2_SCH_3_	-H	1.420 ± 0.028	26.31	13.05	73.8
**41**	-**CH(CH**_**3**_**)**_**2**_	-**CH**_**2**_**(C**_**6**_**H**_**5**_**)**	**-H**	**-0.884 ± 0.036**	**0.13**	**0.05**	**100.0**
**42**	**-CH(CH**_**2**_ **)**_**5**_	-**CH**_**2**_**(C**_**6**_**H**_**5**_**)**	**-H**	**-0.132 ± 0.034**	**0.74**	**0.35**	**100.0**
**43**	-CH(CH_3_)_2_	-CH_2_CH_2_(C_6_H_5_)	-H	1.235 ± 0.048	17.17	13.00	85.4
**44**	-CH(CH_2_)_5_	-CH_2_CH_2_(C_6_H_5_)	-H	1.184 ± 0.046	15.27	7.57	86.5

Among the obtained peptidyl derivatives of α-aminoalkylphosphine oxides with an aliphatic R_3_ substituent, the ability to interact with the BIR3 domain was observed only at high antagonist concentrations (**5**–**8**; Table 1), enabling the determination of EC_50_ values only for the *tert*-butyl derivatives (**5**, **6**). Within the group of derivatives bearing the cyclohexyl ring at R_3_ (**9**, **10**), an interaction with the BIR3 domain was demonstrated by approximately 25% inhibition at the highest antagonist concentration (Table 1). Among compounds that vary at the R_2_ positions and have a fixed R_3_ phenyl ring (**11**–**14**), we observed that introducing a bulky, aromatic R_2_ substituent resulted in an improved interaction with the BIR3 domain. For the R_2_ phosphonic analogs of Gly, the EC_50_ values were 29.33 µM (**11**) and 26.29 µM (**12**), whereas for the Phe analogs, the EC_50_ values decreased significantly, to 1.53 µM and 1.31 µM for R_1_ Val (**13**) and Chg (**14**), respectively. The last group of peptidyl α-aminoalkylphosphine oxides includes derivatives containing small R_2_ (-H or -CH_3_) and aryl R_3_ (-C_6_H_4_-2-CH_3_, -C_6_H_4_-4-CH_3_ or -C_6_H_3_-3,5-(CH_3_)_2_) substituents (**15–22**, Table 1). Within this group, the lowest EC_50_ value was associated with compound **17**, containing R_2_ = -CH_3_ and R_3_ = -C_6_H_4_-4-CH_3_ (EC_50_ value of 19.25 µM).

Among peptidyl derivatives of α-aminoalkylphosphonate diaryl esters (**23**–**28**, Table 2), increasing the size of the phenyl ester ring substituent (R_3_ = -CH_2_CH_3_, -CH(CH_3_)_2_ and -C(CH_3_)_3_) did not significantly change the activity, and EC_50_ values ranged between 19.83 µM (**26**) and 34.25 µM (**23**). The corresponding diphenyl derivatives demonstrated EC_50_ values of 31.86 µM (**29**) and 30.24 µM (**30**). Investigating the effect of R_2_ structure on the ability to interact with the BIR3 domain revealed that among derivatives with an aliphatic R_2_ substituent (**31**–**40**), the Chg-Leu analog had the highest potency (**38**, EC_50_ = 10.61 µM). Similar activity was found for R_2_ Ile derivatives (**35** and **36**, EC_50_ values of 17.84 and 16.24 µM, respectively). Similar to the α-aminoalkylphosphine oxide IAP antagonists, phosphonic Phe analogs were found to be the most potent compounds not only of this group but also of this study, displaying EC_50_ values of 0.13 µM (**41**) and 0.74 µM (**42**). Interestingly, replacing Phe with Hph (**43** and **44**) led to a dramatic decrease in activity compared to parent compounds **41** and **42**, thus highlighting the strong preference toward phenylalanine at this position (Table 2).

**Fig. 5:**
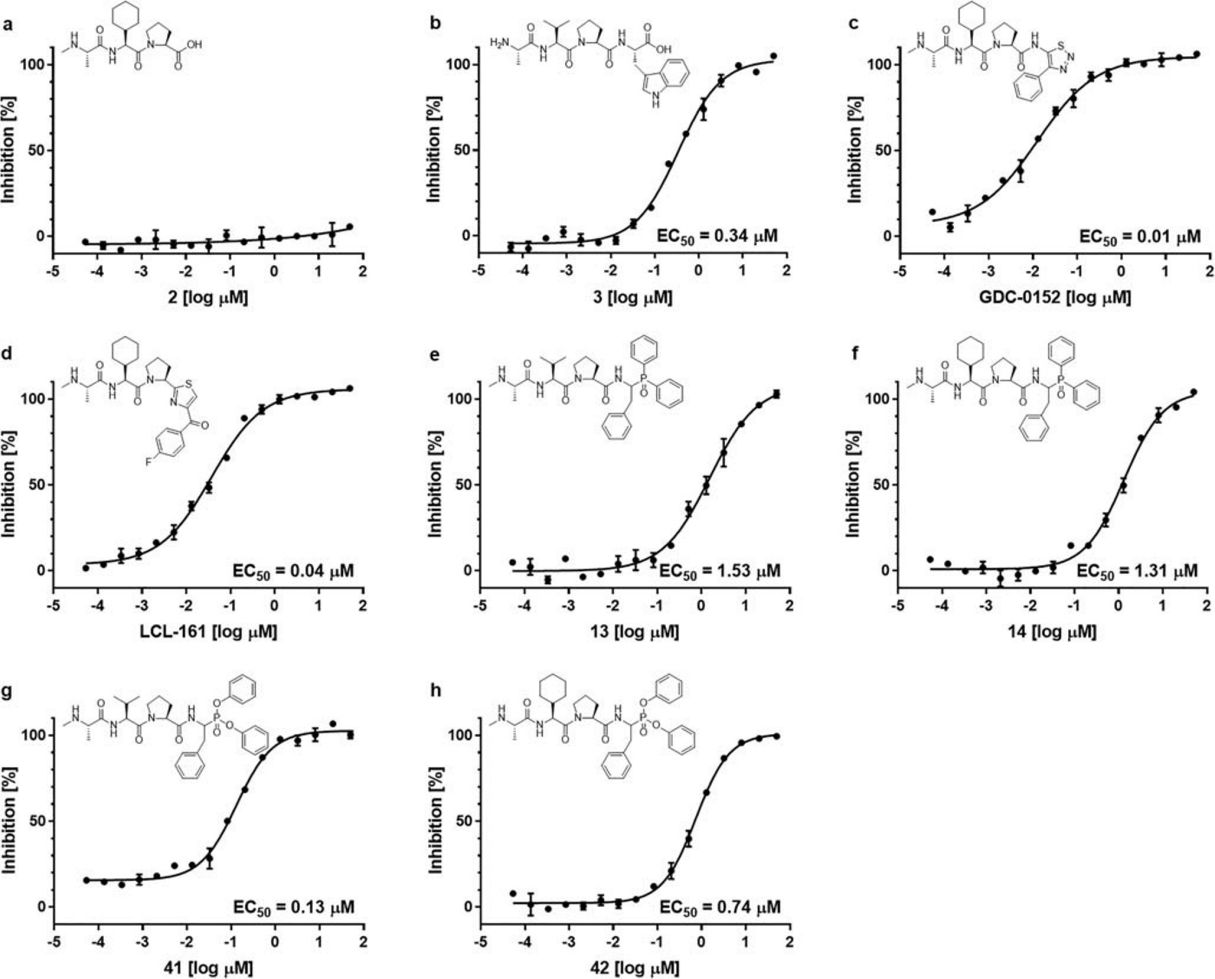
Competitive binding curves obtained for BIR incubated with **2** (negative control, (**a**)), **3** (positive control, (**b**)), reference compounds (**c, d**), and the most potent derivatives obtained in this study (**e-h**)

In summary, the obtained phosphonic peptides represent a novel class of BIR3 antagonists. Among all of the obtained compounds, the highest activity toward the XIAP BIR3 domain was observed for phosphonic Phe analogs, either derivatives of α-aminoalkylphosphine oxides (**13**, **14**) or α-aminoalkylphosphonate diaryl esters (**41**, **42**; Fig. 5). The EC_50_ value for the most potent obtained phosphoroorganic compound (**41**, EC_50_ = 0.13 µM) was more than ten times higher than that of the GDC-0152 reference compound (EC_50_ = 0.01 µM) and almost three and a half times higher than that of LCL-0161 (EC_50_ = 0.04 µM). Nevertheless, in relation to the H_2_N-Ala-Val-Pro-Trp-OH reference tetrapeptide (EC_50_ = 0.34 µM), the EC_50_ value of **41** was over two and a half times lower, indicating the efficacy of **41** when interacting with the XIAP BIR3 domain. Interestingly, replacing Phe with Hph led to decreased BIR domain binding potency. This finding highlights the high preference for the BIR3 domain toward Phe mimetics at this position of the antagonist. It is worth mentioning that the presence of Val or Chg residues in the scaffold seems to have a secondary/less significant role in activity.

**Fig. 6:**
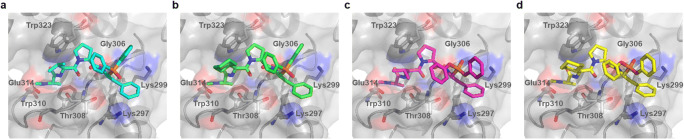
Molecular docking best-scoring models obtained using the Protein-Ligand ANT System (PLANTS v. 1.2) studies of compounds **13** (**a**), **14** (**b**), **41** (**c**) and **42** (**d**). Oxygen, nitrogen, and phosphorus atoms are colored in red, blue and orange, respectively. The solvent accessible surface area of the binding groove of the XIAP protein (1G73.pdb) was made transparent in order to visualize the main residues involved in the interaction with the Ala-Val-Pro-Ile tetrapeptide (stick representation)

In order to examine whether compounds exhibiting activity in the fluorescence polarization assay interact with the binding groove of the XIAP BIR3 domain in a manner analogous to the N-terminal sequence of Smac, we applied *in silico* methods of molecular docking using the Ala-Val-Pro-Ile tetrapeptide as a template. The obtained data showed that the group present at the R_2_ position of the phosphoroorganic compound interacts with the hydrophobic pocket of the BIR3 domain (formed by aliphatic regions of Lys297 and Lys299, Fig. 1) in a similar fashion to the side chain of Ile of the Smac protein when it interacts with the BIR3 domain. The phosphoroorganic moiety together with its substituents is exposed to the solvent (Fig. 6). On the basis of the obtained fluorescence polarization data and molecular docking studies, we showed that phosphoroorganic derivatives of *N*-Me-Ala-Val/Chg-Pro represent a new class of potent IAP antagonists. Further research should focus on detailed structural optimization, as altering the structure of the phosphorus atom substituents of the phenylalanine mimetic might result in the generation of compounds displaying improved potency.

**Fig. 7:**
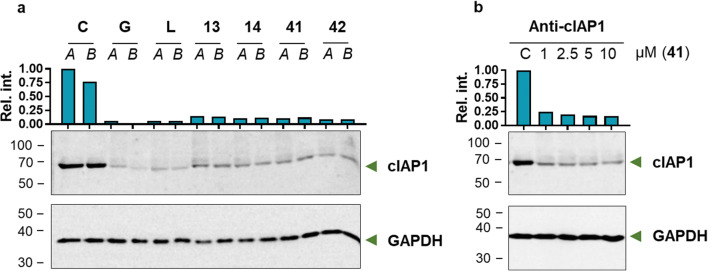
The ability of obtained phosphoroorganic peptide mimetics to induce autoubiquitination and proteasomal degradation of cIAP1. Western blot analysis of MDA-MB-231 cell lysates subjected to incubation with reference compounds: **G** – GDC-0152, **L** – LCL-161, selected phosphoroorganic peptide derivatives (**13**, **14, 41** and **42**) and **C** – negative control treated only with DMSO; *A* and *B* – samples taken after 15 and 120 min incubation with antagonists (**a**). The dose response for compound **41** (1, 2.5, 5 and 10 µM) on the degradation of cIAP1 protein after 15 min incubation with MDA-MB-231 cells; **C** – negative control treated only with DMSO (**b**)

Smac mimetics, in addition to their direct interaction with the BIR domains of the XIAP protein that leads to the liberation of caspases from inhibitor binding, are able to induce autodegradation of the proteins cIAP1 and cIAP2. Autoubiquitination occurs through the activation of E3 ubiquitin ligase activity, which results in proteasome-mediated cIAP degradation [52]. Therefore, we determined whether the obtained phosphoroorganic Smac mimetics were able to induce autoubiquitination followed by cIAP1 degradation. For this purpose, MDA-MB-231 breast cancer cells were incubated either with a phosphoroorganic peptide derivative (**13**, **14**, **41** or **42)** or a reference compound (GDC-0152 or LCL-161). Cell lysates were subjected to Western blot analysis using anti-cIAP1 IgG antibodies (Fig. 7a). The results clearly indicated the ability of the developed derivatives to induce autoubiquitination and degradation of cIAP1 in MDA-MB-231 breast cancer cells. The reference compounds showed slightly higher induction of cIAP1 protein degradation than the developed compounds. Phosphonic Phe analogs (**41** and **42**) showed the greatest ability to induce cIAP1 degradation in the MDA-MB-231 cell line. Because of its high potency of action in the fluorescence polarization assay, compound **41** was subjected to a more detailed, concentration-dependent analysis of its ability to induce cIAP degradation (Fig. 7b and Supplementary Figure S2).

Additionally, we examined the activity of *N*-Me-Ala-Val/Chg-Pro-Phe^P^(OPh)(OH) derivatives (details concerning their structure, synthesis, and kinetic parameters are included in the Supplementary Materials, Supplementary Figure S3). Despite their relatively low EC_50_ values (EC_50_ = 0.43 µM (**41 m**) and 0.52 µM (**42 m**), Supplementary Figure S4) when compared to the corresponding α-aminoalkylphosphine oxides (EC_50_ = 1.53 µM (**13**), EC_50_ = 1.31 µM (**14**)) or diaryl esters of α-aminoalkylphosphonic acids (EC_50_ = 0.13 µM (**41**), EC_50_ = 0.74 µM (**42**)) that demonstrated an effective interaction with the BIR3 domain *via* the fluorescence polarization assay, they were devoid of the autoubiquitination activity and thus did not lead to the degradation of cIAP1 (Supplementary Materials, Supplementary Figure S5). This lack of effectiveness might due to the inability of compounds **41** and **42 m** to cross the cell membrane. This result provides important guidance for further modification of the phosphoroorganic structure for the design of BIR3 domain antagonists.

The in vitro antiproliferative activity of the most promising phosphoroorganic derivatives was evaluated in a set of five diverse human cancer cell lines: two breast cancer cell lines (MDAMB231 and MCF-7), a representative of transitional cell carcinoma (urinary bladder; UMUC3), nonsmall-cell lung carcinoma (A549) and colon adenocarcinoma (LoVo). MCF-10A cells (a nontumorigenic mammary gland epithelial cell line) was used as a reference cell line, whereas GDC0152, LCL161 and cisplatin (CDDP) were used as reference antiproliferative agents. In the case of all XIAP antagonists, the MDAMB231 cell line was significantly more sensitive than any other cell line used. Although the IC_50_ values determined for the commercial antagonists GDC-0152 and LCL-161 were 3–4 times lower (0.88 and 0.76 µM, respectively) than those determined for the selected phosphoroorganic compounds (3.39 µM (**13**), 2.72 µM (**14**), 2.58 µM (**41**), 2.46 µM (**42**)), the antiproliferative potential of our newly described antagonists promotes motivation for further in vitro investigations.

It is notable that no significant differences in IC_50_ values were observed between the tested phosphoroorganic antagonists. The activity of all tested compounds on the MCF7 cell line was limited even at a concentration of 10 µM and was negligible in the remaining cell lines, including the nontumorigenic epithelial cell line MCF10A (Table 3). Such selectivity strongly contrasts with the sensitivity of the tested cell lines for cisplatin – MDAMB231 was the least sensitive cell line with an IC_50_ value several times higher than those for the other cell lines (IC_50_ = 22.14 µM). This distinctive feature of the MDA-MB-231 cell line, which might contribute to the observed selectivity, includes a lack of estrogenic and progesterone receptor expression and amplification of HER2, which are reasons for the wide application of the MDA-MB-231 cell line as a model of poorly differentiated triple negative breast cancer. However, the observed selectivity might also arise from other attributes. The high antiproliferative activity of our novel phosphoroorganic compounds on a cell line that is recognized as a model for chemoresistant, highly aggressive breast cancer combined with a lack of toxicity in a nontumorigenic cell line is an important observation.

**Table 3:** Antiproliferative activity of selected compounds

Compd.	IC_50_ ± SD (*n* = 3) [µM]																		
	**MDA-MB-231**			**MCF-7**			**MCF-10A**			**UMUC-3**			**LoVo**			**A549**			
**GDC-0152**	**0.88** ± 0.21			[18]* ± 4			[1]*> ± 1			[13]* ± 3			[9]* ± 4			[13]* ± 4			
**LCL-161**	**0.76** ± 0.23			[19]* ± 2			[2]* ± 1			[16]* ± 5			[26]* ± 7			[11]* ± 3			
**13**	**3.39** ± 0.42			[22]* ± 6			[5]* ± 2			[6]* ± 2			[6]* ± 2			[2]* ± 1			
**14**	**2.72** ± 0.69			[20]* ± 3			[1]* ± 1			[8]* ± 3			[7]* ± 3			[5]* ± 2			
**41**	**2.58** ± 0.32			[20]* ± 7			[2]* ± 1			[5]* ± 2			[11]* ± 6			[10]* ± 3			
**42**	**2.46** ± 0.38			[16]* ± 2			[2]* ± 1			[6]* ± 3			[12]* ± 4			[23]* ± 6			
**CDDP**	**22.14** ± 5.26			**5.36** ± 1.8			**6.76** ± 1.2			**3.73** ± 0.63			**3.9** ± 1.3			**1.6** ± 0.4			

* - mean proliferation inhibition [%] at highest concentration used (10 µM)

**Fig. 8:**
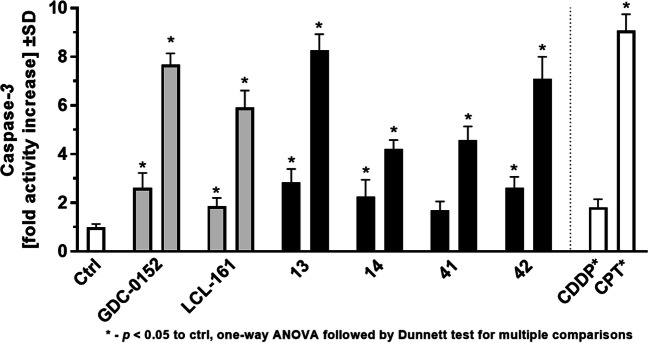
Potential of selected compounds as apoptosis inducers measured *via* caspase-3 enzymatic activity assay after 24 h of exposure. For GDC-0152 and LCL-161, the left bar corresponds to the sample treated with a concentration of 0.1 µM, and the right bar corresponds to the sample treated with a concentration of 1 µM. For selected phosphoroorganic peptide derivatives (**13**, **14, 41** and **42**), the left bar corresponds to 1 µM, and the right bar corresponds to 10 µM. CDDP and CPT were applied at a concentration of 10 µM

Further studies on the proapoptotic potential of the developed compounds showed that they could induce strong caspase3 activity in MDA-MB-231 cells, with an activity comparable to that induced by camptothecin (CPT) when used at a concentration of 10 µM (1 µM for GDC-0152 and LCL-161). Both the reference XIAP BIR3 domain antagonists and the newly synthesized derivatives were indicated to be significantly more potent than cisplatin when used at 10 µM (1 µM for GDC-0152 and LCL-161) and at least equally active when used at 1 µM (0.1 µM for GDC-0152 and LCL-161; cisplatin was used at a fixed concentration of 10 µM) (Fig. 8).

In conclusion, the designed and synthesized derivatives represent the first example of phosphorus-containing antagonists of the XIAP BIR3 domain. The observed selective antiproliferative activity associated with strong apoptosis induction might open an avenue for further development of novel, low molecular weight IAP antagonists that might be useful as potential anticancer agents.

## Materials and methods

### General

All chemical reagents and solvents were obtained from commercial sources and used without purification. High-resolution mass spectra (HRMS) were acquired on Waters Acquity Ultra Performance LC, LCT Premier/XE (Waters, Warszawa, Poland) or Bruker micrOTOF-QII (Bruker, Poznań, Poland) spectrometers. The nuclear magnetic resonance spectra (^1^H and ^31^P) were recorded on either a 400 MHz NMR Jeol PCZ 400S (Jeol, Warszawa, Poland) or 600 MHz NMR Bruker Avance spectrometer (Bruker). High-performance liquid chromatography analysis and purification were carried out on a Varian ProStar system (Varian, Australia) or a Waters Binary Module System (Waters,) with a dual λ absorbance detector system using a Discovery BIO Wide Pore C8 HPLC column (250 mm × 21.2 mm, 10 µm) with a 15 mL/min flow rate using a linear gradient from 0 to 100% B within 20 min or a Discovery BIO Wide Pore C8 HPLC column (250 mm × 4.6 mm, 10 µm) with a 1 mL/min flow rate using a linear gradient from 0 to 100% B over 15 min (solvent A: H_2_O with 0.05% trifluoroacetic acid, solvent B: MeCN with 0.05% trifluoroacetic acid).

### Docking studies

Molecular docking studies of the designed IAP protein phosphoroorganic antagonists were performed using the crystal structure of the XIAP BIR3 domain as a receptor (1G73.pdb) and the tetrapeptide H_2_N-Ala-Val-Pro-Ile-OH as a template whose structure was a pattern of constraints used for the docked antagonists. The geometry of phosphoroorganic compounds **13**, **14**, **41** and **42** was optimized with the MM2 force field (as implemented in ChemBio3D Ultra 11.0). Atom types and structural protonation were set with SPORES [53]. Docking studies were carried out by means of the Protein-Ligand ANT System (PLANTS ver. 1.2) [54] with an IAP peptide-binding groove defined in the binding site with a 20 Å radius and a center corresponding to the C_α_ of Leu307.

### Chemistry

#### Synthesis of the peptides

Peptides *N*-Me-Ala-Val-Pro-OH and *N*-Me-Ala-Chg-Pro-OH were synthesized manually on solid support using the Fmoc strategy. For this purpose, 2-Cl-Trt resin (1.6 mmol/g) was preloaded with Fmoc-Pro-OH (1.2 eq.). The resulting level of substitution (0.88 mmol/g) was determined spectrophotometrically at 300 nm after deprotection with 30% piperidine in dimethylformamide (DMF). During peptide synthesis, the Fmoc group was removed by means of 20% piperidine in DMF, while Fmoc-protected amino acids (3 eq.) were subsequently introduced with 2-(1H-benzotriazol-1-yl)-1,1,3,3-tetramethyluronium hexafluorophosphate (HBTU) as a coupling agent (3 eq.) in the presence of *N,N*-diisopropylethylamine (DIPEA, 5 eq.). The *N*-terminal methyl group was introduced into the peptide on the resin *via* the Mitsunobu reaction [47]. Briefly, the *N*-terminal Fmoc protecting group was removed with 20% piperidine in DMF, followed by a 15 min incubation with 4-nitrobenzenesulfonyl chloride (*o*-NBS-Cl, 4 eq.) and collidine (10 eq.) in NMP. Next, the resin was washed with NMP and THF followed by the addition of MeOH (10 eq.), Ph_3_P (5 eq.) and DIAD (5 eq.) in THF. The reaction was performed at r.t. for 30 min, and the resin was washed with THF and NMP. Removal of the *o*-NBS group was performed using HSCH_2_CH_2_OH (10 eq.) and 1,8-diazabicyclo[5.4.0]undec-7-ene (DBU, 5 eq.) in THF (2 × 10 min). Cleavage of the obtained *N*-methylated tripeptides (*N*-Me-Ala-Val-Pro-OH (**1**) and *N*-Me-Ala-Chg-Pro-OH (**2**)) from the resin was achieved by means of a solution of TFA:TIPS:H_2_O (95:2.5:2.5, *v/v/v*; 2 h, r.t.). The obtained peptides were purified with HPLC and analyzed by HRMS and NMR.

The tetrapeptide H_2_N-Ala-Val-Pro-Trp-OH (**3**) was synthesized on a solid support (2-Cl-Trt resin; 1.6 mmol/g) using the Fmoc strategy and HBTU as a coupling agent described above, starting with Fmoc-Trp(^*t*^Boc)-OH. The peptide cleavage from the resin was performed with TFA:TIPS:H_2_O solution (95:2.5:2.5, *v/v/v*; 2 h, r.t.), followed by HPLC purification and HRMS and NMR analysis.

#### Synthesis of the α-aminoalkylphosphine oxides

Tritylamine was synthesized from trityl chloride and ammonium chloride in a 25% ammonium hydroxide solution. Briefly, to an ice bath-cooled solution of ammonium chloride (0.2 mol) in ammonium hydroxide (200 mL), trityl chloride (0.2 mol) dissolved in toluene (200 mL) was added dropwise over 2 h. The reaction continued overnight at r.t. with vigorous stirring. The organic layer was washed with water until a neutral pH was reached, dried over sodium sulfate and evaporated under vacuum to dryness, yielding the final product as a white solid (94%) [55].

Tritylimines were obtained by reacting tritylamine (1 eq.) with different aldehydes (4 eq.): formaldehyde, acetaldehyde, phenylacetaldehyde or 2-methylbutyraldehyde in anhydrous toluene (for 24 h at r.t.). The progress of the reaction was monitored by TLC. The reaction mixture was washed with water, dried over anhydrous sodium sulfate and evaporated to dryness, yielding the target tritylimines that were used in the next step without purification.

**Fig. 9:**
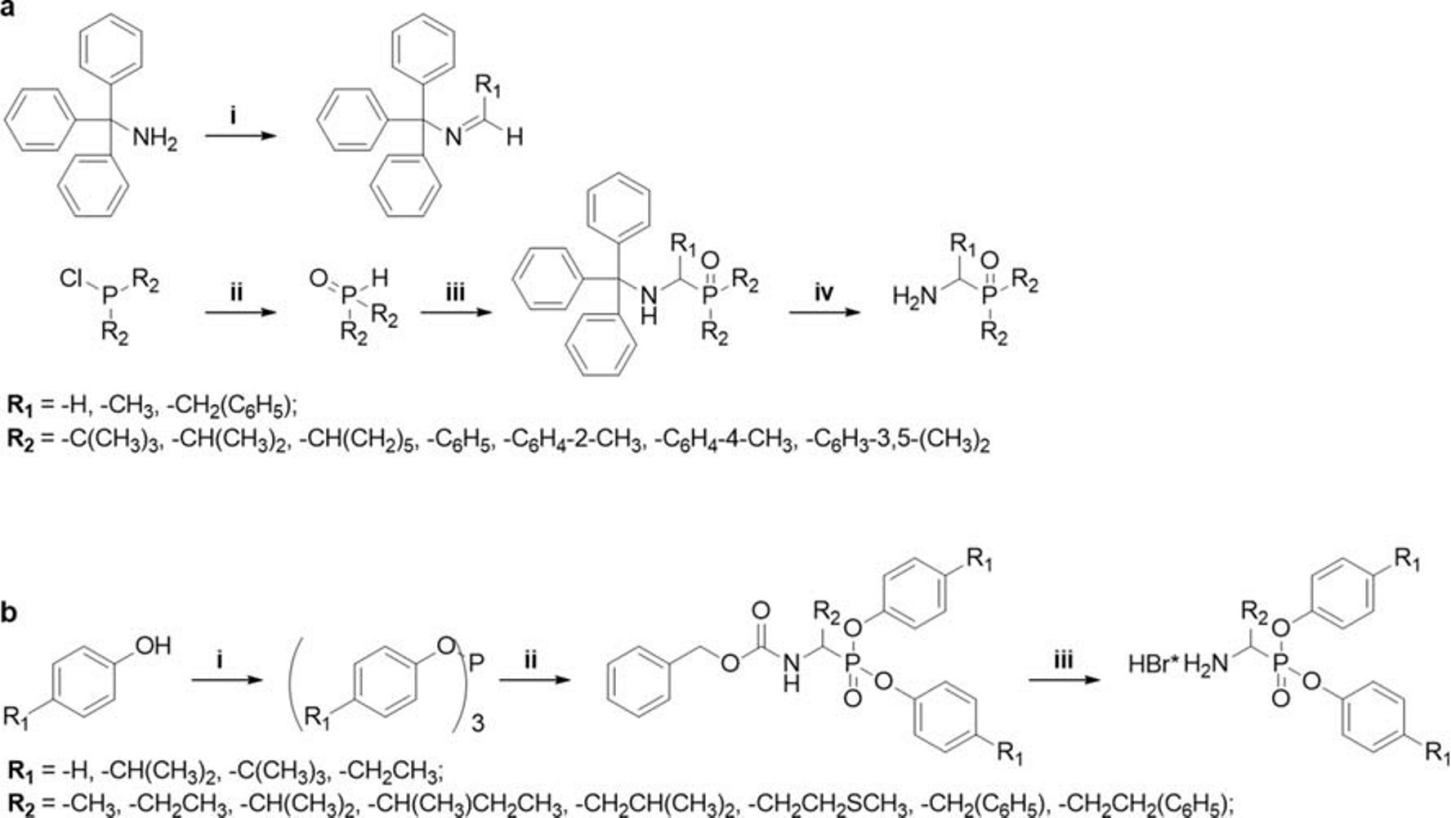
Synthesis of phosphoroorganic derivatives. The synthesis of α-aminoalkylphosphine oxides: (**i**) R_1_-CHO, toluene, r.t.; (**ii**) 1 M HCl, r.t.; (**iii**) Trt-N = CHR_1_, toluene, 8 h, reflux; (**iv**) TFA, r.t. or HCl/MeOH, reflux (**a**). The synthesis of α-aminoalkylphosphonate diaryl esters: (**i**) PCl_3_, acetonitrile, 4 h, reflux; (**ii**) Cbz-NH_2_, R_2_-CHO, AcOH, 4 h, reflux; (**iii**) 33% HBr/AcOH, 2 h, r.t. (**b**)

The synthesis of α-aminoalkylphosphine oxides started with the preparation of diaryl or dialkyl phosphine oxides obtained *via* the oxidation of diaryl or dialkyl chlorophosphines with 1 M HCl_aq_ (0 °C → r.t., 24 h). The resulting crude phosphine oxide (1 eq.) was dissolved in toluene, and a tritylimine (1 eq.) was added. The reaction was performed under reflux for 8 h. The volatile components were removed under vacuum, and the trityl protecting group was removed with TFA (2 h, r.t.) for the aryl derivatives or HCl (10 eq.) in MeOH (30 min, reflux) for the alkyl derivatives. The crude products were used in the next steps of peptidyl derivative preparation. The synthesis of the α-aminoalkylphosphine oxides is outlined in Fig. 9a.

#### Synthesis of the α-aminoalkylphosphonate diaryl esters

The synthesis started with the preparation of triaryl phosphites, which were obtained from the appropriate phenol (3 eq.) and PCl_3_ (1 eq.) in refluxing acetonitrile [56]. The α-amidoalkylation reaction of the obtained triaryl phosphite with benzyl carbamate (1.1 eq.) and an aldehyde (1.1 eq.) was performed in glacial acetic acid (80–90 °C, 2 h) [57]. After evaporation under reduced pressure, the resulting oil was dissolved in methanol, and the product crystallized at -20 °C. The Cbz protecting group was removed with 33% HBr/AcOH (2 h, r.t.) and the product crystallized in diethyl ether as an HBr salt. The synthesis pathway of α-aminoalkylphosphonate diaryl esters is shown in Fig. 9b.

#### Peptide scaffold modification with phosphoroorganic functionality

Peptide scaffolds (**1** and **2**) were coupled with α-aminoalkylphosphine oxides or α-aminoalkylphosphonate diaryl esters (1.2 eq.), using HBTU as a coupling agent (1.2 eq.) in the presence of DIPEA (5 eq.) in MeCN or DMSO. The reaction was performed overnight at r.t. The solvent was removed in vacuo, and the target compounds were isolated directly using HPLC, followed by spectroscopic analysis.

#### Synthesis of the fluorescent probe

Synthesis of the fluorescein-labeled peptide was performed on solid support applying the Fmoc strategy (Rink Amide resin; 0.6 mmol/g) using HBTU (3 eq.) and DIPEA (3 eq.) for amino acid coupling in DMF. Fmoc deprotection was performed with 20% piperidine in DMF. In the resulting resin-bound peptide, Boc-Ala-Val-Pro-Dpm-Ala-Lys(Mtt)-Lys(Boc)-resin, the Mtt group was removed with 1% TFA solution in DCM. The progress of Mtt removal was monitored spectrophotometrically at 460 nm [50]. Subsequently, the resin-bound peptide was labeled with 5(6)-carboxyfluorescein (5(6)-Fam, 2.2 eq.) using HATU as a coupling agent (3 eq.) and HOBt as an additive (3 eq.) in the presence of DIPEA (3.5 eq.). The obtained fluorescent probe was cleaved off of the resin with a TFA:TIPS:H_2_O solution (95:2.5:2.5, *v/v/v*, 2 h, r.t.) yielding H-Ala-Val-Pro-Dpm-Ala-Lys(5(6)-Fam)-Lys-NH_2_ (**4**), which was purified by HPLC and analyzed spectroscopically.

### Fluorescence polarization assay

The fluorescence polarization assay was performed at r.t. in Corning 384-well black, polystyrene flat bottom plates (Corning, NY, USA) on a Synergy H4 Hybrid Reader (BioTek, Bad Friedrichshall, Germany). For fluorescence polarization measurements, (milipolarization units, mP) 484/20 nm excitation and 528/20 nm emission filters were selected. Phosphate buffer (pH 7.5) supplemented with γ-globulin (100 µg/mL) and NaN_3_ (0.02% *w/v*), fluorescent probe **4** and the XIAP BIR3 domain (Sino Biological, Beijing, China) were used for all measurements. All experiments were performed in duplicate. The obtained fluorescence polarization assay data were analyzed with Prism 5.0 Software (GraphPad Software, San Diego, ca.).

#### Binding assay

In order to determine the dissociation constant (K_d_) between the XIAP BIR3 domain and fluorescent probe **4**, serial dilutions of the XIAP BIR3 domain (two-fold dilutions, ranging from 2.5 µM to 0.0763 nM) were incubated with the probe (2 nM) for 30 min. Fluorescence polarization values were plotted as a function of the protein concentration, and the K_d_ value was determined using GraphPad software (*one site - total binding model*).

#### Inhibition assay

For all phosphoroorganic peptide derivatives, the inhibition constant values were determined by the addition of the XIAP BIR3 protein (50 nM; established experimentally based on the K_d_ value) into the phosphoroorganic compound solution (5-fold dilutions, ranging from 50 µM to 0.640 nM for screening purposes and 2.5-fold dilutions ranging from 50 µM to 0.0537 nM for detailed analysis). After a 15 min incubation at r.t., fluorescent probe **4** (2 nM) was added. The values ​​were measured 30 min after the moment the probe was added. The assay was performed in phosphate buffer with a final DMSO concentration of 1.4% (*v/v*). As a reference, inhibitors, tetrapeptide H-Ala-Val-Pro-Trp-OH (**3**) and commercially available IAP antagonists (GDC-0152 and LCL-161) were used. In order to evaluate the quality and precision of the performed screening assay, we determined the Z´ factor as described by Zhang et al. [58]. The obtained fluorescence polarization values were used to determine the percent inhibition, which was plotted as a function of the protein concentration. The EC_50_ and K_i_ values were calculated with GraphPad software (*log(agonist) vs. response (variable slope) model*) in combination with the web tool developed by Nikolovska-Coleska et al. [59].

### Biological activity of selected peptide derivatives

#### Cell culture

MDA-MB-231 (human breast cancer), MCF10A (human, nontumorigenic mammary gland epithelial cells), A549 (human nonsmall-cell lung cancer), UMUC-3 (human bladder cancer) and LoVo (human colon cancer) cell lines were obtained from the American Type Culture Collection (ATCC; Rockville, Maryland, USA). The MCF-7 cell line (human breast cancer) was obtained from the European Collection of Cell Cultures (ECACC; Salisbury, UK). All cell lines were maintained at the Hirszfeld Institute of Immunology and Experimental Therapy (HIIET), Wrocław, Poland. MDA-MB-231 cells were cultured in RPMI1640 medium (Thermo Fisher Scientific, Warszawa, Poland) supplemented with 2 mM l-glutamine, 1 mM sodium pyruvate, and 10% fetal bovine serum (all from Sigma-Aldrich, Poznań, Poland). The MCF10A cell line was cultured in Ham’s F12 medium with glutamine (Corning) supplemented with 5% (*v*/*v*) fetal bovine serum (FBS), 5% (*v*/*v*) horse serum, 10 µg/mL insulin, 0.05 µg/mL cholera toxin, 0.5 µg/mL hydrocortisone, and 20 ng/mL hEGF (all from Sigma-Aldrich). A549 and LoVo cells were cultured in a 1:1 (*v*/*v*) mixture of RPMI1640 and Opti-MEM (both from HIIET) supplemented with 5% (*v*/*v*) FBS and 2 mM L-glutamine. The UMUC-3 cell line was cultured in high glucose DMEM (Thermo Fisher Scientific) supplemented with 10% FBS and 2 mM Lglutamine. The MCF-7 line was cultured in Eagle’s minimal essential medium (EMEM; Thermo Fisher Scientific) supplemented with 10% (*v*/*v*) FBS, 2 mM L-glutamine, 1% (*v*/*v*) nonessential amino acids, and 8 µg/mL insulin (all from Sigma-Aldrich). All culture media contained 100 µg/mL streptomycin (Sigma-Aldrich) and 100 U/mL penicillin (Polfa Tarchomin SA, Warszawa, Poland). The cells were grown at 37 °C in a humid atmosphere saturated with 5% CO_2_.

#### Western blot analysis

Prior to Western blot sample collection, cells were harvested by trypsinization, counted and seeded on 50 mm Petri dishes. After overnight incubation, the culture medium was replaced with fresh medium containing the tested compounds at the target concentration ranges with 0.1% (*v/v*) DMSO as a control. Next, the cells were washed with PBS and lysed with ice-cold RIPA buffer containing protease and phosphatase inhibitors (Sigma-Aldrich, Poznań, Poland), and after debris removal (4 °C, 15 min, 16,000 × g), the lysates were used in further experiments.

MDA-MB-231 cell lysates (25 µg of total protein/lane) were resolved by SDS-PAGE (4–12%, Tris-glycine, reducing conditions) and transferred onto a nitrocellulose membrane (0.45 µm pore size; Thermo Scientific, Gdańsk, Poland) using the semi-dry blotting system (Cleaver Scientific, Rugby, UK). The membrane was washed with 10 mM PBS (pH 7.4; 5 min, r.t.) and blocked with 5% skim milk in PBS supplemented with 0.05% Tween-20 (PBST) for 1 h at r.t. Subsequently, the membrane was washed with PBST (3 times for 5 min, r.t.) and incubated with rabbit anti-cIAP1 monoclonal IgG (1:1,000 in 0.5% skim milk in PBST; 4 °C, overnight; Cell Signaling Technology, Warszawa, Poland, Cat# 7065, D5G9). Next, the membrane was washed with PBST (3 times for 5 min, r.t.) and incubated with goat anti-rabbit IgG-HRP (1:1,000 in 0.5% skim milk in PBST; Sigma-Aldrich, Cat# A6154). After incubation (1 h, r.t.), the membrane was washed with PBST (3 times for 5 min, r.t.), and chemiluminescent peroxidase substrate was added (Super Signal WestPico, Thermo Scientific). The bands were visualized by means of the blot imaging system (GelLogic 1500, Carestream, Rochester, NY, USA).

For the loading control, the membrane was washed with PBST and incubated with mouse anti-GAPDH IgG (1:1,000 in 0.5% skim milk in PBST; Thermo Scientific, Cat# MA515738, GA1R). After incubation (1 h, 37 °C), the membrane was washed with PBST (3 times for 5 min, r.t.) and incubated with anti-mouse HRP-labeled rabbit IgG (1:5,000 in 0.5% skim milk in PBST; Fitzgerald, Acton, MA, USA, Cat# 43R-1424) for 1 h at 37 °C. After washing, the signal was developed as described above.

#### Antiproliferative activity analysis

Antiproliferative activity was assessed in six cell lines utilizing the sulforhodamine B (SRB) method [60]. Briefly, cells were seeded in 96-well plates (Sarstedt, Warszawa, Poland) at a density of 5 × 10^3^ cells/well, and after overnight incubation, the cells were exposed to compounds at various concentrations. After 72 h, the plates were fixed with 50% (*v*/*v*) TCA (all from Sigma-Aldrich), washed with tap water, and the precipitated proteins were labeled with 0.1% (*w*/*v*) SRB solution in 1% (*v*/*v*) AcOH. Next, the plates were washed with 1% (*v*/*v*) AcOH, and the remaining SRB dye was solubilized with 10 mM unbuffered TRIS solution. The absorbance was measured using a Biotek Hybrid H4 reader (BioTek Instruments, VT, USA) at 540 nm. Crude results were analyzed using a previously described method [61], and the results are expressed as the IC_50_ (compound concentration that reduced cell growth by 50%) compared to the vehicletreated control (0.1% (*v*/*v*) DMSO). The samples were analyzed in triplicate, and each experiment was repeated at least three times.

#### Caspase-3 activity assay for apoptosis rate analysis

Caspase-3 activity was assessed in MB-MB-231 cells utilizing a previously described protocol [62] with minor modifications. Briefly, cells were seeded in 48-well plates at a density of 20 × 10^3^ cells/well, cultured overnight and treated with compounds at various concentrations for 24 h. Then, the cells were lysed by applying ice-cold lysis buffer. Next, the samples were transferred to an opaque 96-well plate (Corning) with reaction buffer that contained Ac-DEVD-AMC (a caspase3 fluorogenic substrate; Cayman Chemicals, Ann Arbor, USA). Sample fluorescence was continuously recorded at 37 °C for 2 h using a Biotek Synergy H4 Hybrid Reader (λ_ex_ 350 nm, λ_em_ 460 nm). Crude results for maximal velocity were normalized to the protein content using the SRB method and are reported as a caspase-3 activity fold increase compared to the vehicletreated control (0.1% (*v*/*v*) DMSO).

## Electronic supplementary material

Additional data generated or analyzed during this study is included in the Supplementary Materials files.

ESM 1 (PDF 658 kb)
