# Supplementary material for: Structure-based design, synthesis, and evaluation of the biological activity of novel phosphoroorganic small molecule IAP antagonists
Source: Invest New Drugs. 2020 Apr 8;38(5):1350–64. doi: 10.1007/s10637-020-00923-4 (PMC7497679; doi:10.1007/s10637-020-00923-4)
Supplement: Supplementary file 1 — (PDF 658 kb) [file 10637_2020_923_MOESM1_ESM.pdf]

Structure-based design, synthesis, and evaluation of the biological activity of novel phosphoroorganic small molecule IAP antagonists

Agnieszka Łupicka-Słowik[1], Mateusz Psurski[2], Renata Grzywa[1], Monika Cuprych[2], Jarosław Ciekot[2], Waldemar Goldeman[1], Elżbieta Wojaczyńska[3], Jacek Wojaczyński[4], Józef Oleksyszyn[1], Marcin Sieńczyk[*,1]

[1] Faculty of Chemistry, Department of Organic and Medicinal Chemistry, Wrocław University of Science and Technology, Wybrzeże Wyspiańskiego 27, 50-370 Wrocław, Poland.
[2] Department of Experimental Oncology, Hirszfeld Institute of Immunology and Experimental Therapy, Polish Academy of Sciences, Weigla 12, 53-114, Wrocław, Poland
[3] Faculty of Chemistry, Department of Physical and Quantum Chemistry, Wrocław University of Science and Technology, Wybrzeże Wyspiańskiego 27, 50-370 Wrocław, Poland.
[4] Department of Chemistry, University of Wrocław, F. Joliot-Curie 14, 50-383, Wrocław, Poland.

[*]Author for correspondence:
Tel.: +48 71 320 36 46
Fax: +48 71 320 24 27
marcin.sienczyk@pwr.edu.pl
ORCID: 0000-0002-5528-5264

Supplementary materials

Details concerning the composition of obtained products……………………………………………..Page 2

Stability of the free and bound fluorescent probe in fluorescence polarization assay, determination of the Z′ factor……………………………………………………………………...……Page 9

The ability of the compound ***41*** *to induce autoubiquitination and proteasomal degradation of cIAP1…………………………………………………...…….….……Page 10*

Synthesis of aryl monoester of α-amino-methylphenyl-phosphonic acid as a phosphoroorganic derivative of N-Me-Ala-Val/Chg-Pro-OH tripeptide………………….Page 11

Potency of synthesized phosphoroorganic peptide derivatives ***41m*** *and* ***42m*** *to interact with binding groove of the BIR3 domain……………………………………….……..Page 12*

Western blot analysis of the potential of ***41m*** *to induce rapid autoubiquitination and proteasome degradation of cIAP1 in MDA-MB-231 breast cancer cells…….….……….Page 13*

Details concerning molecular weights and composition of obtained products.

(S)-1-((S)-3-methyl-2-((S)-2-(methylamino)propanamido)butanoyl)pyrrolidine-2-carboxylic acid (**1**). ^{1}H NMR (400 MHz, CD_3OD): δ 0.99 (d, J = 8.0 Hz, 3H), 1.05 (d, J = 8.0 Hz, 3H), 1.45 (d, J = 8.0 Hz, 3H), 1.93-2.15 (m, 4H), 2.20-2.28 (m, 1H), 2.64 (s, 3H), 3.66-3.72 (m, 1H), 3.84-3.90 (m, 2H), 4.38 (dd, J = 8.0, 4.0 Hz, 1H), 4.48 (d, J = 8.0 Hz, 1H). HRMS: calcd for $(C_{14}H_{25}N_3O_4)H^+$, 300.1923; found, 300.1911.

(S)-1-((S)-2-cyclohexyl-2-((S)-2-(methylamino)propanamido)acetyl)pyrrolidine-2-carboxylic acid (**2**). ^{1}H NMR (400 MHz, CD_3OD): δ 1.01-1.33 (m, 6H), 1.44 (d, J = 8.0 Hz, 3H), 1.65-1.85 (m, 6H), 1.91-2.06 (m, 4H), 2.18-2.29 (m, 1H), 2.63 (s, 3H), 3.66-3.72 (m, 1H), 3.84-3.91 (m, 2H), 4.36 (dd, J = 8.0, 4.0 Hz, 1H), 4.49 (d, J = 8.0 Hz, 1H). HRMS: calcd for $(C_{17}H_{29}N_3O_4)H^+$, 340.2236; found, 340.2239.

(S)-2-((S)-1-((S)-2-((S)-2-aminopropanamido)-3-methylbutanoyl)pyrrolidine-2-carboxamido)-3-(1H-indol-3-yl)propanoic acid (**3**). ^{1}H NMR (400 MHz, CD_3OD): δ 0.86 (d, J = 8.0 Hz, 3H), 0.93 (d, J = 8.0 Hz, 3H), 1.43 (d, J = 8.0 Hz, 3H), 1.85-2.04 (m, 4H), 2.07-2.16 (m, 1H), 3.20-3.25 (m, 1H), 3.30-3.39 (m, 1H), 3.51-3.56 (m, 1H), 3.75-3.80 (m, 1H), 3.93-3.97 (m, 1H), 4.40-4.45 (m, 2H), 4.67-4.70 (m, 1H), 6.95-7.08 (m, 2H), 7.14 (s, 1H), 7.30 (d, J = 8.0 Hz, 1H), 7.57 (d, J = 8.0 Hz, 1H). HRMS: calcd for $(C_{24}H_{33}N_5O_5)H^+$, 472.2560; found, 472.2549. Yield 39%.

(S)-1-(L-alanyl-L-valyl)-N-((S)-1-(((S)-1-(((S)-1-(((S)-1,6-diamino-1-oxohexan-2-yl)amino)-6-(3',6'-dihydroxy-3-oxo-3H-spiro[isobenzofuran-1,9'-xanthene]-5-carboxamido)-1-oxohexan-2-yl)amino)-1-oxopropan-2-yl)amino)-1-oxo-3,3-diphenylpropan-2-yl)pyrrolidine-2-carboxamide (4). ^{1}H NMR (400 MHz, DMSO-d_6): δ 0.84 (d, J = 8.0 Hz, 3H), 0.88 (d, J = 8.0 Hz, 3H), 0.98-1.06 (m, 3H), 1.16-1.28 (m, 8H), 1.39-1.54 (m, 7H), 1.57-1.72 (m, 3H), 1.76-1.85 (m, 1H), 1.89-1.99 (m, 1H), 2.65-2.76 (m, 2H), 2.79-2.86 (m, 1H), 3.46-3.58 (m, 4H), 3.80-3.91 (m, 1H), 4.01-4.15 (m, 3H), 4.17-4.22 (m, 1H), 4.26-4.31 (m, 1H), 4.37-4.44 (m, 1H), 5.10-5.20 (m, 1H), 6.49-6.54 (m, 3H), 6.64-6.68 (m, 1H), 6.99-7.36 (m, 11H), 7.61-7.70 (m, 3H), 7.76-7.85 (m, 2H), 7.97-8.06 (m, 2H), 8.12-8.23 (m, 1H), 8.41-8.47 (m, 1H). HRMS: calcd for $(C_{64}H_{76}N_{10}O_{13})H^+$, 1193.5666; found, 1193.5953. Yield 12%.

(S)-N-((di-tert-butylphosphoryl)methyl)-1-((S)-3-methyl-2-((S)-2-(methylamino)propanamido)butanoyl)pyrrolidine-2-carboxamide (**5**). ^{1}H NMR (400 MHz, $CDCl_3$): δ 0.91 (d, J = 8.0 Hz, 3H), 0.99 (d, J = 8.0 Hz, 3H), 1.21 (d, J = 8.0 Hz, 9H), 1.25 (d, J = 8.0 Hz, 9H), 1.47 (d, J = 8.0 Hz, 3H), 1.90-2.22 (m, 6H), 2.79 (s, 3H), 3.66-3.72 (m, 2H), 3.74-3.80 (m, 2H), 4.35 (dd, J = 16.0, 8.0 Hz, 1H), 4.55-4.63 (m, 2H), 8.06 (bs, 1H), 8.24 (d, J = 8.0 Hz, 1H). ^{31}P NMR (162 MHz, $CDCl_3$): δ 62.57 (s). HRMS: calcd for $(C_{23}H_{45}N_4O_4P)H^+$, 473.3251; found, 473.3247. Yield 29%.

(S)-1-((S)-2-cyclohexyl-2-((S)-2-(methylamino)propanamido)acetyl)-N-((di-tert-butylphosphoryl)methyl)pyrrolidine-2-carboxamide (**6**). ^{1}H NMR (400 MHz, $CDCl_3$): δ 0.94-1.04 (m, 3H), 1.09 (s, 1H), 1.20 (d, J = 4.0 Hz, 9H), 1.24 (d, J = 4.0 Hz, 9H), 1.42 (d, J = 8.0 Hz, 3H), 1.65-1.83 (m, 6H), 1.92-2.00 (m, 2H), 2.11-2.20 (m, 2H), 2.81 (s, 3H), 3.38-3.46 (m, 1H), 3.65-3.75 (m, 2H), 3.77-3.85 (m, 2H), 4.05-4.15 (m, 1H), 4.41-4.46 (m, 1H), 4.52 (dd, J = 8.00, 4.00 Hz, 1H), 4.59 (t, J = 8.00 Hz, 1H), 8.26 (bs, 1H), 8.43 (d, J = 8.00 Hz, 1H). ^{31}P NMR (162 MHz, $CDCl_3$): δ 62.30 (s). HRMS: calcd for $(C_{26}H_{49}N_4O_4P)H^+$, 513.3570; found, 513.3567. Yield 53%.

(S)-N-((diisopropylphosphoryl)methyl)-1-((S)-3-methyl-2-((S)-2-(methylamino)propanamido)butanoyl)pyrrolidine-2-carboxamide (**7**). ^{1}H NMR (400 MHz, $CDCl_3$): δ 0.91 (d, J = 8.0 Hz, 3H), 1.00 (d, J = 8.0 Hz, 3H), 1.12-1.22 (m, 12H), 1.42 (d, J = 4.0 Hz, 3H), 1.85-2.25 (m, 8H), 2.78 (s, 3H), 3.50-3.57 (m, 1H), 3.67-3.88 (m, 3H), 4.29-4.34 (m, 1H), 4.49-4.53 (m, 1H), 4.61 (t, J = 8.0 Hz, 1H), 8.28 (bs, 1H), 8.47 (bs, 1H). ^{31}P NMR (162 MHz, $CDCl_3$): δ 59.28 (s). HRMS: calcd for $(C_{21}H_{41}N_4O_4P)H^+$, 445.2938; found, 445.2932. Yield 47%.

(S)-1-((S)-2-cyclohexyl-2-((S)-2-(methylamino)propanamido)acetyl)-N-((diisopropylphosphoryl)methyl)pyrrolidine-2-carboxamide (**8**). ^{1}H NMR (400 MHz, $CDCl_3$): δ 1.14-1.23 (m, 16H), 1.37 (d, J = 8.0 Hz, 3H), 1.67-1.85 (m, 6H), 1.91-1.99 (m, 2H), 2.04-2.20 (m, 4H), 2.78 (s, 3H), 3.44-3.51 (m, 1H), 3.88-3.96 (m, 5H), 4.32 (dd, J = 8.0, 4.0 Hz, 1H), 4.49 (dd, J = 4.0, 4.0 Hz, 1H), 4.59 (t, J = 8.0 Hz, 1H), 8.41 (bs, 1H), 8.52 (d, J = 8.0 Hz, 1H). ^{31}P NMR (162 MHz, $CDCl_3$): δ 59.88 (s). HRMS: calcd for $(C_{24}H_{45}N_4O_4P)H^+$, 485.3299; found, 485.3253. Yield 25%.

(S)-N-((dicyclohexylphosphoryl)methyl)-1-((S)-3-methyl-2-((S)-2-(methylamino)propanamido)butanoyl)pyrrolidine-2-carboxamide (**9**). ^{1}H NMR (400 MHz, $CDCl_3$): δ 0.91 (d, J = 8.0 Hz, 3H), 1.02 (d, J = 8.0 Hz, 3H), 1.17-1.35 (m, 13H), 1.40 (d, J = 8.0 Hz, 3H), 1.75-1.99 (m, 12H), 2.03-2.13 (m, 2H), 2.19-2.26 (m, 1H), 2.78 (s, 3H), 3.47-3.51 (m, 1H), 3.67-3.73 (m, 1H), 3.77-3.84 (m, 2H), 4.35 (dd, J = 16.0, 8.0 Hz,1H), 4.49-4.52 (m, 1H), 4.63 (t, J = 8.0 Hz, 1H), 8.30 (bs, 1H), 8.54 (d, J = 8.0 Hz, 1H). ^{31}P NMR (162 MHz, $CDCl_3$): δ 53.39 (s). HRMS: calcd for $(C_{27}H_{49}N_4O_4P)H^+$, 525.3564; found, 525.3561. Yield 51%.

(S)-1-((S)-2-cyclohexyl-2-((S)-2-(methylamino)propanamido)acetyl)-N-((dicyclohexylphosphoryl)methyl)pyrrolidine-2-carboxamide (**10**). ^{1}H NMR (400 MHz, $CDCl_3$): δ 0.86-1.34 (m, 18H), 1.39 (d, J = 8.0 Hz, 3H), 1.63-1.95 (m, 18H), 2.01-2.23 (m, 2H), 2.71 (s, 3H), 3.58-3.77 (m, 4H), 4.11 (dd, J = 8.0, 4.0 Hz, 1H), 4.48-4.55 (m, 2H), 8.13 (bs, 1H), 8.35 (bs, 1H). ^{31}P NMR (162 MHz, $CDCl_3$): δ 53.41 (s). HRMS: calcd for $(C_{30}H_{53}N_4O_4P)H^+$, 565.3883; found, 565.3880. Yield 69%.

(S)-N-((diphenylphosphoryl)methyl)-1-((S)-3-methyl-2-((S)-2-(methylamino)propanamido)butanoyl)pyrrolidine-2-carboxamide (**11**). ^{1}H NMR (400 MHz, $CDCl_3$): δ 0.91 (d, J = 8.0 Hz, 3H), 0.97 (d, J = 8.0 Hz, 3H), 1.36 (d, J = 8.0 Hz, 3H), 1.79-1.95 (m, 3H), 2.06-2.13 (m, 1H), 2.69-2.91 (m, 5H), 3.60-3.65 (m, 1H), 3.69-3.74 (m, 1H), 3.80-3.95 (m, 1H), 4.28-4.39 (m, 2H), 4.40-4.55 (m, 1H), 4.65 (t, J = 8.0 Hz, 1H), 7.41-7.63 (m, 8H), 7.74-7.78 (m, 2H), 8.61 (bs, 1H), 9.05 (bs, 1H). ^{31}P NMR (162 MHz, $CDCl_3$): δ 33.77 (s). HRMS: calcd for $(C_{27}H_{37}N_4O_4P)H^+$, 513.2631; found, 513.2612. Yield 69%.

(S)-1-((S)-2-cyclohexyl-2-((S)-2-(methylamino)propanamido)acetyl)-N-((diphenylphosphoryl)methyl)pyrrolidine-2-carboxamide (**12**) ^{1}H NMR (400 MHz, $CDCl_3$): δ 1.01-1.17 (m, 7H), 1.37 (d, J = 8.0 Hz, 3H), 1.45-2.01 (m, 10H), 2.72 (s, 3H), 3.60-3.65 (m, 1H), 3.70-3.76 (m, 1H), 3.82-3.93 (m, 1H), 4.26-4.31 (m, 1H), 4.38-4.41 (m, 1H), 4.46-4.61 (m, 3H), 7.42-7.65 (m, 8H), 7.74-7.79 (m, 2H), 8.52 (bs, 1H), 9.20 (bs, 1H). ^{31}P NMR (162 MHz, $CDCl_3$): δ 33.53 (s). HRMS: calcd for $(C_{30}H_{41}N_4O_4P)H^+$, 553.2944; found, 553.2956. Yield 69%.

(2S)-N-(1-(diphenylphosphoryl)-2-phenylethyl)-1-((S)-3-methyl-2-((S)-2-(methylamino)propanamido)butanoyl)pyrrolidine-2-carboxamide (**13**). ^{1}H NMR (400 MHz, $CDCl_3$): δ 0.71 (d, J = 6.8 Hz, 3H), 0.81 (d, J = 6.4 Hz, 3H), 1.58 (d, J = 7.2 Hz, 3H), 1.69-1.86 (m, 5H), 2.87 (s, 3H), 2.90-2.97 (m, 2H), 3.15-3.23 (m, 1H), 3.32-3.41 (m, 1H), 3.55-3.64 (m, 1H), 4.35-4.43 (m, 1H), 4.47-4.54 (m, 1H), 5.29-5.36 (m, 1H), 7.14-7.91 (m, 15H), 8.14 (bs, 1H), 8.41 (s, 1H), 9.34 (d, J = 9.6 Hz, 1H). ^{31}P NMR (162 MHz, $CDCl_3$): δ 35.50 (s). HRMS: calcd for $(C_{34}H_{43}N_4O_4P)H^+$, 603.3095; found, 603.3091. Yield 45%.

(2S)-1-((S)-2-cyclohexyl-2-((S)-2-(methylamino)propanamido)acetyl)-N-(1-(diphenylphosphoryl)-2-phenylethyl)pyrrolidine-2-carboxamide (**14**). ^{1}H NMR (400 MHz, $CDCl_3$): δ 0.98-1.18 (m, 6H), 1.58 (d, J = 6.8 Hz, 3H), 1.61-1.80 (m, 8H), 2.13-2.22 (m, 1H), 2.57 (s, 3H), 2.89-2.99 (m, 2H), 3.14-3.24 (m, 1H), 3.49-3.67 (m, 2H), 4.28-4.52 (m, 2H), 5.25-5.33 (m, 1H), 7.12-7.61

(m, 15H), 8.29 (bs, 1H), 8.49 (s, 1H), 9.30 (d, *J* = 9.6 Hz, 1H). ^{31}P NMR (162 MHz, $CDCl_3$): δ 35.45 (s). HRMS: calcd for $(C_{37}H_{47}N_4O_4P)H^+$, 643.3408; found, 643.3405. Yield 35%.

(S)-N-((di-p-tolylphosphoryl)methyl)-1-((S)-3-methyl-2-((S)-2-(methylamino)propanamido)butanoyl)pyrrolidine-2-carboxamide (**15**). ^{1}H NMR (400 MHz, $CDCl_3$): δ 0.91 (d, *J* = 8.0 Hz, 3H), 0.97 (d, *J* = 8.0 Hz, 3H), 1.09 (s, 1H), 1.37 (d, *J* = 8.0 Hz, 3H), 1.80-2.13 (m, 5H), 2.36 (d, *J* = 8.0 Hz, 6H), 2.73 (s, 3H), 3.60-3.66 (m, 1H), 3.69-3.75 (m, 1H), 3.78-3.90 (m, 1H), 4.28-4.41 (m, 3H), 4.65 (dd, *J* =8.4, 7.2 Hz, 1H), 7.18-7.23 (m, 2H), 7.27-7.32 (m, 2H), 7.40-7.50 (m, 2H), 7.55-7.65 (m, 2H), 8.58 (bs, 1H), 9.06 (bs, 1H). ^{31}P NMR (162 MHz, $CDCl_3$): δ 34.14 ppm. HRMS: calcd for $(C_{29}H_{41}N_4O_4P)H^+$, 541.2944; found, 541.2918. Yield 50%.

(S)-1-((S)-2-cyclohexyl-2-((S)-2-(methylamino)propanamido)acetyl)-N-((di-p-tolylphosphoryl)methyl)pyrrolidine-2-carboxamide (**16**). ^{1}H NMR (400 MHz, $CDCl_3$): δ 0.95-1.19 (m, 6H), 1.38 (d, *J* = 7.2 Hz, 3H), 1.54-1.85 (m, 8H), 1.88-1.98 (m, 2H), 2.36 (s, 3H), 2.38 (s, 3H), 2.73 (s, 3H), 3.61-3.67 (m, 1H), 3.69-3.76 (m, 1H), 3.80-3.93 (m, 1H), 4.25-4.45 (m, 3H), 4.58-4.64 (m, 1H), 7.21-7.23 (m, 2H), 7.28-7.31 (m, 2H), 7.44-7.49 (m, 2H), 7.58-7.63 (m, 2H), 8.51 (bs, 1H), 9.19 (bs, 1H). ^{31}P NMR (162 MHz, $CDCl_3$): δ 39.93 ppm. HRMS: calcd for $(C_{32}H_{45}N_4O_4P)H^+$, 581.3257; found, 581.3251. Yield 51%.

(2S)-1-((S)-2-cyclohexyl-2-((S)-2-(methylamino)propanamido)acetyl)-N-(1-(di-p-tolylphosphoryl)ethyl)pyrrolidine-2-carboxamide (**17**). ^{1}H NMR (400 MHz, $CDCl_3$): δ 1.09-1.24 (m, 9H), 1.36-1.71 (m, 8H), 1.73-1.90 (m, 4H), 1.91-2.02 (m, 1H), 2.35 (s, 3H), 2.36 (s, 3H), 2.73 (d, *J* = 51.2 Hz, 3H), 3.38-3.50 (m, 1H), 3.63-3.72 (m, 1H), 4.06-4.13 (m, 1H), 4.24-4.36 (m, 1H), 4.48-4.67 (m, 1H), 5.02-5.14 (m, 1H), 7.20-7.30 (m, 4H), 7.47-7.69 (m, 4H), 8.45 (bs, 1H), 8.80 (bs, 1H). ^{31}P NMR (243 MHz, DMSO-d_6): δ 31.63 (s, 51%), 32.20 (s, 49%). HRMS: calcd for $(C_{33}H_{47}N_4O_4P)H^+$, 595.3395; found, 595.3408. Yield 44%.

(S)-N-((di-o-tolylphosphoryl)methyl)-1-((S)-3-methyl-2-((S)-2-(methylamino)propanamido)butanoyl)pyrrolidine-2-carboxamide (**18**). ^{1}H NMR (400 MHz, $CDCl_3$): δ 0.85 (d, *J* = 8.0 Hz, 3H), 0.96 (d, *J* = 8.0 Hz, 3H), 1.25 (d, *J* = 8.0 Hz, 3H), 1.59-1.71 (m, 1H), 1.83-1.92 (m, 1H), 1.99-2.10 (m, 3H), 2.24 (s, 3H), 2.25 (s, 3H), 2.63 (s, 3H), 2.91 (s, 1H) 3.63-3.78 (m, 3H), 3.98-4.10 (m, 1H), 4.18-4.23 (m, 1H), 4.50 (dd, *J* = 8.0 Hz, 1H), 4.62 (dd, *J* = 7.6 Hz, 1H), 7.10-7.35 (m, 4H), 7.36-7.50 (m, 2H), 7.67-7.75 (m, 2H), 8.67 (bs, 1H), 8.94 (bs, 1H). ^{31}P NMR (162 MHz, $CDCl_3$): δ 36.15 ppm. HRMS: calcd for $(C_{29}H_{41}N_4O_4P)H^+$, 541.2944; found, 541.2950. Yield 62%.

(S)-1-((S)-2-cyclohexyl-2-((S)-2-(methylamino)propanamido)acetyl)-N-((di-o-tolylphosphoryl)methyl)pyrrolidine-2-carboxamide (**19**). ^{1}H NMR (400 MHz, $CDCl_3$): δ 0.95-1.18 (m, 6H), 1.24 (s, 3H), 1.55-1.91 (m, 8H), 1.95-2.06 (m, 1H), 2.27 (s, 3H), 2.28 (s, 3H), 2.60 (s, 3H), 3.27 (bs, 1H), 3.64-3.77 (m, 3H), 3.85-4.05 (m, 1H), 4.06-4.18 (m, 1H), 4.52 (dd, *J* = 7.2, 4.8 Hz, 1H), 4.59 (dd, *J* = 7.2 Hz, 1H), 7.17-7.30 (m, 4H), 7.40-7.46 (m, 2H), 7.64-7.74 (m, 2H), 8.55 (bs, 1H), 9.13 (bs, 1H). ^{31}P NMR (162 MHz, $CDCl_3$): δ 36.13 ppm. HRMS: calcd for $(C_{32}H_{45}N_4O_4P)H^+$, 581.3257; found, 581.3252. Yield 54%.

(S)-N-((bis(3,5-dimethylphenyl)phosphoryl)methyl)-1-((S)-3-methyl-2-((S)-2-(methylamino)propanamido)butanoyl)pyrrolidine-2-carboxamide (**20**). ^{1}H NMR (400 MHz, $CDCl_3$): δ 0.89 (d, *J* = 6.4 Hz, 3H), 0.98 (d, *J* = 6.8 Hz, 3H), 1.38 (d, *J* = 6.8 Hz, 3H),1.80-1.89 (m, 1H), 1.91-2.00 (m, 1H), 2.03-2.12 (m, 3H), 2.28 (s, 6H), 2.33 (s, 6H), 2.76 (s, 3H), 3.60-3.66 (m, 1H), 3.69-3.75 (m, 1H), 3.97-4.08 (m, 1H), 4.11-4.19 (m, 1H), 4.32-4.40 (m, 2H), 4.48 (dd, *J* = 8.0, 6.0 Hz, 1H), 4.65 (dd, *J* = 7.2 Hz, 1H), 7.12-7.29 (m, 6H), 8.58 (bs, 1H), 8.91 (d, *J* = 7.6 Hz, 1H). ^{31}P NMR (162

MHz, $CDCl_3$): δ 33.74 ppm. HRMS: calcd for $(C_{31}H_{45}N_4O_4P)H^+$, 569.3257; found, 569.3264. Yield 63%.

(S)-N-((bis(3,5-dimethylphenyl)phosphoryl)methyl)-1-((S)-2-cyclohexyl-2-((S)-2-(methylamino)propanamido)acetyl)pyrrolidine-2-carboxamide (**21**). ^1H NMR (400 MHz, $CDCl_3$): δ 0.95-1.17 (m, 5H), 1.37 (d, J = 6.8 Hz, 3H), 1.51-1.88 (m, 10H), 2.01-2.09 (m, 1H), 2.29 (s, 6H), 2.33 (s, 6H), 2.76 (s, 3H), 3.61-3.68 (m, 1H), 3.70-3.75 (m, 1H), 3.89-4.05 (m, 1H), 4.10-4.24 (m, 1H), 4.28-4.36 (m, 1H), 4.48 (dd, J = 8.4, 6.4 Hz, 1H), 4.61 (dd, J = 8.0 Hz, 1H), 7.13 (s, 1H), 7.17 (s, 1H), 7.18 (s, 1H), 7.22 (s, 1H), 7.27 (s, 1H), 7.31 (s, 1H), 8.51 (bs, 1H), 9.05 (bs, 1H). ^{31}P NMR (162 MHz, $CDCl_3$): δ 33.67 ppm. HRMS: calcd for $(C_{34}H_{49}N_4O_4P)H^+$, 609.3574; found, 609.3564. Yield 28%.

(2S)-N-(1-(bis(3,5-dimethylphenyl)phosphoryl)ethyl)-1-((S)-3-methyl-2-((S)-2-(methylamino)propanamido)butanoyl)pyrrolidine-2-carboxamide (**22**). ^1H NMR (400 MHz, $CDCl_3$): δ 0.91-0.97 (m, 6H), 1.13-1.18 (m, 3H), 1.28-1.33 (m, 3H), 1.68-2.13 (m, 5H), 2.25-2.35 (m, 12H), 2.77 (s, 3H), 3.35-3.49 (m, 1H), 3.64-3.72 (m, 1H), 4.10-4.31 (m, 2H), 4.36-4.44 (m, 1H), 4.54-4.68 (m, 1H), 5.04-5.16 (m, 1H), 7.11 (s, 1H), 7.12 (s, 1H), 7.27-7.40 (m, 4H), 8.38 (bs, 1H), 8.86 (bs, 1H). ^{31}P NMR (162 MHz, $CDCl_3$): δ 37.70 (s, 49%), 37.88 (s, 51%). HRMS: calcd for $(C_{32}H_{47}N_4O_4P)H^+$, 583.3406; found, 583.3408. Yield 32%.

bis(4-ethylphenyl) (1-((S)-1-((S)-3-methyl-2-((S)-2-(methylamino)propanamido)butanoyl)pyrrolidine-2-carboxamido)ethyl)phosphonate (**23**). ^1H NMR (400 MHz, $CDCl_3$): δ 0.84 (d, J = 6.8 Hz, 3H), 0.97 (d, J = 6.0 Hz, 3H), 1.14-1.21 (m, 9H), 1.40-1.51 (m, 3H), 1.74-2.10 (m, 5H), 2.55-2.61 (m, 7H), 3.65-3.80 (m, 3H), 4.10-4.20 (m, 1H), 4.45-4.52 (m, 1H), 4.63 (dd, J = 8.0 Hz, 1H), 4.70-4.86 (m, 1H), 6.98-7.12 (m, 8H), 8.23 (d, J = 8.4 Hz, 1H), 8.69 (bs, H). ^{31}P NMR (243 MHz, DMSO-d_6): δ 19.55 (s, 67%), 19.95 (s, 33%). HRMS: calcd for $(C_{32}H_{47}N_4O_6P)H^+$, 615.3311; found, 615.3334. Yield 46%.

bis(4-ethylphenyl) (1-((S)-1-((S)-2-cyclohexyl-2-((S)-2-(methylamino)propanamido)acetyl)pyrrolidine-2-carboxamido)ethyl)phosphonate (**24**). ^1H NMR (400 MHz, $CDCl_3$): δ 0.99-1.28 (m, 17H), 1.38-1.54 (m, 5H), 1.82-2.08 (m, 5H), 2.48-2.64 (m, 7H), 3.65-3.75 (m, 1H), 3.77-3.85 (m, 1H), 4.11-4.23 (m, 1H), 4.43-4.51 (m, 1H), 4.55-4.65 (m, 1H), 4.73-4.85 (m, 1H), 6.96-7.12 (m, 8H), 8.25 (bs, 1H), 8.75 (bs, 1H), 8.83 (bs, 1H). ^{31}P NMR (243 MHz, DMSO-d_6): δ 19.53 (s, 60%), 19.97 (s, 40%). HRMS: calcd for $(C_{35}H_{51}N_4O_6P)H^+$, 655.3624; found, 655.3640. Yield 46%.

bis(4-isopropylphenyl) (1-((S)-1-((S)-3-methyl-2-((S)-2-(methylamino)propanamido)butanoyl)pyrrolidine-2-carboxamido)ethyl)phosphonate (**25**). ^1H NMR (400 MHz, CD_3OD): δ 0.84 (d, J = 7.2 Hz, 3H), 0.99 (d, J = 7.2 Hz, 3H), 1.18 (d, J = 7.2 Hz, 12H), 1.26 (d, J = 6.8 Hz, 3H), 1.40-1.51 (m, 3H), 1.73-2.13 (m, 5H), 2.61 (s, 3H), 2.81-2.89 (m, 2H), 3.66-3.72 (m, 1H), 3.75-3.81 (m, 1H), 4.16-4.24 (m, 1H), 4.46-4.52 (m, 1H), 4.62-4.67 (m, 1H), 4.73-4.84 (m, 1H), 6.98-7.19 (m, 8H), 8.24 (d, J = 8.8 Hz, 1H), 8.63-8.69 (m, 1H), 8.71-8.75 (m, 1H). ^{31}P NMR (243 MHz, DMSO-d_6): δ 19.54 (s, 70%), 19.96 (s, 30%). HRMS: calcd for $(C_{34}H_{51}N_4O_6P)H^+$, 643.3624; found, 643.3642. Yield 40%.

bis(4-isopropylphenyl) (1-((S)-1-((S)-2-cyclohexyl-2-((S)-2-(methylamino)propanamido)acetyl)pyrrolidine-2-carboxamido)ethyl)phosphonate (**26**). ^1H NMR (400 MHz, $CDCl_3$): δ 0.86-1.12 (m, 5H), 1.17-1.27 (m, 18H), 1.56-1.62 (m, 2H), 1.69-1.78 (m, 3H), 1.82-2.11 (m, 5H), 2.60 (s, 3H), 2.81-2.89 (m, 2H), 3.66-3.75 (m, 1H), 3.78-3.83 (m, 1H), 4.16-4.22 (m, 1H), 4.48 (dd, J = 8.0, 4.0 Hz, 1H), 4.59-4.65 (m, 1H), 4.72-4.86 (m, 1H), 6.94-7.16 (m, 8H), 8.25 (d, J = 8.4 Hz, 1H), 8.75 (bs, 1H), 8.84 (d, J = 8.8 Hz, 1H). ^{31}P NMR (243 MHz, DMSO-d_6): δ 19.52 (s, 60%), 19.99 (s, 40%). HRMS: calcd for $(C_{37}H_{55}N_4O_6P)H^+$, 683.3937; found, 683.3960. Yield 34%.

bis(4-(tert-butyl)phenyl) (1-((S)-1-((S)-3-methyl-2-((S)-2-(methylamino)propanamido)butanoyl)pyrrolidine-2-carboxamido)ethyl)phosphonate (**27**). ^1H NMR (400 MHz, $CDCl_3$): δ 0.84 (d, J = 6.0 Hz, 3H), 0.99 (d, J = 6.4 Hz, 3H), 1.26-1.28 (m, 24H), 1.81-2.11 (m, 5H), 2.62 (s, 3H), 3.67-3.71 (m, 1H), 3.75-3.81 (m, 1H), 4.17-4.23 (m, 1H), 4.50 (dd, J = 5.6 Hz, 1H), 4.64 (dd, J = 8.4 Hz, 1H), 4.72-4.84 (m, 1H), 6.99-7.11 (m, 4H), 7.27-7.33 (m, 4H), 8.21 (d, J = 9.6 Hz, 1H), 8.62 (d, J = 8.0 Hz, 1H), 8.72 (d, J = 8.0 Hz, 1H). ^{31}P NMR (243, MHz, DMSO-d_6): δ 19.51 (s, 70%), 19.96 (s, 30%). HRMS: calcd for $(C_{36}H_{55}N_4O_6P)H^+$, 671.3937; found, 671.3942. Yield 44%.

bis(4-(tert-butyl)phenyl) (1-((S)-1-((S)-2-cyclohexyl-2-((S)-2-(methylamino)propanamido)acetyl)pyrrolidine-2-carboxamido)ethyl)phosphonate (**28**). ^1H NMR (400 MHz, $CDCl_3$): δ 0.85-1.18 (m, 5H), 1.24-1.28 (m, 24H), 1.55-1.80 (m, 5H), 1.83-2.12 (m, 5H), 2.60 (s, 3H), 3.66-3.74 (m, 1H), 3.78-3.83 (m, 1H), 4.16-4.21 (m, 1H), 4.48 (dd, J = 6.0, 4.8 Hz, 1H), 4.61 (dd, J = 8.4 Hz, 1H), 4.70-4.85 (m, 1H), 6.95-7.11 (m, 4H), 7.26-7.33 (m, 4H), 8.23 (d, J = 7.6 Hz, 1H), 8.71 (bs, 1H), 8.82 (d, J = 6.8 Hz, 1H). ^{31}P NMR (243 MHz, DMSO-d_6): δ 19.51 (s, 61%), 19.98 (s, 39%). HRMS: calcd for $(C_{39}H_{59}N_4O_6P)H^+$, 711.4250; found, 711.4264. Yield 50%.

diphenyl (1-((S)-1-((S)-3-methyl-2-((S)-2-(methylamino)propanamido)butanoyl)pyrrolidine-2-carboxamido)ethyl)phosphonate (**29**). ^1H NMR (400 MHz, $CDCl_3$): δ 0.84 (d, J = 5.6 Hz, 3H), 1.00 (d, J = 6.8 Hz, 3H), 1.23-1.27 (m, 3H), 1.45-1.53 (m, 3H),), 1.81-2.12 (m, 5H), 2.61 (s, 3H), 3.66-3.74 (m, 1H), 3.76-3.85 (m, 1H), 4.15-4.26 (m, 1H), 4.47-4.53 (m, 1H), 4.61-4.66 (m, 1H), 4.72-4.88 (m, 1H), 7.08-7.17 (m, 6H), 7.27-7.30 (m, 4H), 8.30 (d, J = 9.6 Hz, 1H), 8.71 (d, J = 8.0 Hz, 1H), 8.78 (d, J = 9.3 Hz, 1H). ^{31}P NMR (243 MHz, DMSO-d_6): δ 19.50 (s, 64%), 19.88 (s, 36%). HRMS: calcd for $(C_{28}H_{39}N_4O_6P)H^+$, 559.2685; found, 559.2678. Yield 41%.

diphenyl (1-((S)-1-((S)-2-cyclohexyl-2-((S)-2-(methylamino)propanamido)acetyl)pyrrolidine-2-carboxamido)ethyl)phosphonate (**30**). ^1H NMR (400 MHz, $CDCl_3$): δ 0.95-1.14 (m, 5H), 1.23 (d, J = 3.2 Hz, 3H), 1.45-1.55 (m, 3H), 1.57-1.81 (m, 5H), 1.83-1.98 (m, 3H), 2.02-2.12 (m, 2H), 2.60 (s, 3H), 3.66-3.73 (m, 1H), 3.79-3.85 (m, 1H), 4.14-4.22 (m, 1H), 4.46-4.50 (m, 1H), 4.59-4.65 (m, 1H), 4.69-4.88 (m, 1H), 7.05-7.19 (m, 6H), 7.27-7.34 (m, 4H), 8.32 (s, J = 8.4 Hz, 1H), 8.81 (d, J = 7.6 Hz, 1H), 8.87 (d, J = 8.4 Hz, 1H). ^{31}P NMR (243 MHz, DMSO-d_6): δ 19.50 (s, 60%), 19.90 (s, 40%). HRMS: calcd for $(C_{31}H_{43}N_4O_6P)H^+$, 599.2993; found, 599.2995. Yield 44%.

diphenyl (1-((S)-1-((S)-3-methyl-2-((S)-2-(methylamino)propanamido)butanoyl)pyrrolidine-2-carboxamido)propyl)phosphonate (**31**). ^1H NMR (400 MHz, $CDCl_3$): δ 0.82 (d, J = 5.6 Hz, 3H), 0.99-1.03 (m, 6H), 1.23 (d, J = 7.6 Hz, 3H), 1.65-2.15 (m, 7H), 2.59 (s, 3H), 3.70-3.88 (m, 2H), 4.20-4.35 (m, 2H), 4.53-4.71 (m, 2H), 7.07-7.18 (m, 6H), 7.24-7.30 (m, 4H), 8.18 (d, J = 8.8 Hz, 1H), 8.60 (d, J = 5.2 Hz, 1H), 8.79 (d, J = 7.2 Hz, 1H). ^{31}P NMR (243 MHz, DMSO-d_6): δ 18.77 (s, 61%), 19.13 (s, 39%). HRMS: calcd for $(C_{29}H_{41}N_4O_6P)H^+$, 573.2836; found, 573.2838. Yield 43%.

diphenyl (1-((S)-1-((S)-2-cyclohexyl-2-((S)-2-(methylamino)propanamido)acetyl)pyrrolidine-2-carboxamido)propyl)phosphonate (**32**). ^1H NMR (400 MHz, CD_3OD): δ 0.86-1.24 (m, 12H), 1.49-2.12 (m, 11H), 2.58 (s, 3H), 3.71-3.80 (m, 1H), 3.82-3.93 (m, 1H), 4.20-4.29 (m, 1H), 4.50-4.77 (m, 3H), 7.06-7.31 (m, 10H), 8.29 (bs, 1H), 8.55 (bs, 1H), 8.94 (s, 1H). ^{31}P NMR (243 MHz, DMSO-d_6): δ 18.72 (s, 64%), 19.09 (s, 36%). HRMS: calcd for $(C_{32}H_{45}N_4O_6P)H^+$, 613.3149; found, 613.3150. Yield 50%.

diphenyl (2-methyl-1-((S)-1-((S)-3-methyl-2-((S)-2-(methylamino)propanamido)butanoyl)pyrrolidine-2-carboxamido)propyl)phosphonate (**33**). ^1H NMR (400 MHz, $CDCl_3$): δ 0.80 (d, J = 5.2 Hz, 3H), 0.98-1.03 (m, 3H), 1.05-1.15 (m, 6H), 1.19-1.30

(m, 3H), 1.83-2.13 (m, 5H), 2.60 (s, 3H), 3.72-3.92 (m, 2H), 4.26-4.33 (m, 1H), 4.56-4.76 (m, 3H), 7.06-7.19 (m, 6H), 7.22-7.35 (m, 4H), 8.13 (d, *J* = 8.4 Hz, 1H), 8.51 (d, *J* = 6.8 Hz, 1H), 8.81 (bs, 1H). ^{31}P NMR (243 MHz, DMSO-d_6): δ 18.37 (s, 58%), 18.72 (s, 42%). HRMS: calcd for $(C_{30}H_{43}N_4O_6P)H^+$, 587.2993; found, 587.2991. Yield 37%.

diphenyl (1-((S)-1-((S)-2-cyclohexyl-2-((S)-2-(methylamino)propanamido)acetyl)pyrrolidine-2-carboxamido)-2-methylpropyl)phosphonate (**34**). ^{1}H NMR (400 MHz, CDCl$_3$): δ 1.00 (d, *J* = 6.4 Hz, 3H), 1.06-1.11 (m, 8H), 1.16-1.20 (m, 3H), 1.45-1.83 (m, 5H), 1.87-2.13 (m, 4H), 2.25-2.42 (m, 2H), 2.58 (s, 3H), 3.74-3.83 (m, 1H), 3.86-3.96 (m, 1H), 4.24-4.39 (m, 1H), 4.54-4.78 (m, 3H), 7.07-7.20 (m, 6H), 7.25-7.32 (m, 4H), 8.27 (d, *J* = 8.4 Hz, 1H), 8.46 (bs, 1H), 8,97 (bs, 1H). ^{31}P NMR (243 MHz, DMSO-d_6): δ 18.31 (s, 60%), 18.69 (s, 40%). HRMS: calcd for $(C_{33}H_{47}N_4O_6P)H^+$, 627.3306; found, 627.3302. Yield 29%.

diphenyl (2-methyl-1-((S)-1-((S)-3-methyl-2-((S)-2-(methylamino)propanamido)butanoyl)pyrrolidine-2-carboxamido)butyl)phosphonate (**35**). ^{1}H NMR (400 MHz, CDCl$_3$): δ 0.82-0.90 (m, 7H), 0.98-1.12 (m, 8H), 1.25-1.31 (m, 3H), 1.83-2.10 (m, 5H), 2.61 (s, 3H), 3.68-3.93 (m, 2H), 4.23-4.40 (m, 1H), 4.55-4.68 (m, 2H), 4.71-4.96 (m, 1H), 7.09-7.18 (m, 6H), 7.26-7.31 (m, 4H), 7.96-8.06 (m, 1H), 8.40-8.53 (m, 1H), 8.61-8.80 (m, 1H). ^{31}P NMR (243 MHz, DMSO-d_6): δ 18.62 (s, 20%), 18.81 (s, 21%), 18.87 (s, 35%), 19.14 (s, 24%). HRMS: calcd for $(C_{31}H_{45}N_4O_6P)H^+$, 601.3149; found, 601.3152. Yield 31%.

diphenyl (1-((S)-1-((S)-2-cyclohexyl-2-((S)-2-(methylamino)propanamido)acetyl)pyrrolidine-2-carboxamido)-2-methylbutyl)phosphonate (**36**). ^{1}H NMR (400 MHz, CDCl$_3$): δ 0.80-0.91 (m, 5H), 0.98-1.11 (m, 9H), 1.20 (t, J = 6.0 Hz, 3H), 1.66-1.82 (m, 4H), 1.85-2.15 (m, 6H), 2.59 (s, 3H), 3.73-3.81 (m, 1H), 3.85-3.97 (m, 1H), 4.22-4.39 (m, 1H), 4.52-4.67 (m, 2H), 4.70-4.99 (m, 1H), 7.07-7.19 (m, 6H), 7.27-7.31 (m, 4H), 8.16-8.24 (m, 1H), 8.33-8.47 (m, 1H), 8.85-8.92 (m, 1H). ^{31}P NMR (243 MHz, DMSO-d_6): δ 18.59 (s, 21%), 18.76 (s, 20%), 18.83 (s, 34%), 19.08 (s, 25%). HRMS: calcd for $(C_{34}H_{49}N_4O_6P)H^+$, 641.3462; found, 641.3472. Yield 34%.

diphenyl (3-methyl-1-((S)-1-((S)-3-methyl-2-((S)-2-(methylamino)propanamido)butanoyl)pyrrolidine-2-carboxamido)butyl)phosphonate (**37**). ^{1}H NMR (400 MHz, CDCl$_3$): δ 0.85-1.02 (m, 12H), 1.19-1.24 (m, 5H), 1.83-2.07 (m, 6H), 2.61 (s, 3H), 3.72-3.89 (m, 2H), 4.20-4.29 (m, 1H), 4.54-4.63 (m, 2H), 4.74-4.86 (m, 1H), 7.06-7.17 (m, 6H), 7.23-7.29 (m, 4H), 8.13 (d, *J* = 7.2 Hz, 1H), 8.55 (d, *J* = 8.4 Hz, 1H), 8.75-8.86 (m, 1H). ^{31}P NMR (243 MHz, DMSO-d_6): δ 19.32 (s, 70%), 19.61 (s, 30%). HRMS: calcd for $(C_{31}H_{45}N_4O_6P)H^+$, 601.3155; found, 601.3149. Yield 44%.

diphenyl (1-((S)-1-((S)-2-cyclohexyl-2-((S)-2-(methylamino)propanamido)acetyl)pyrrolidine-2-carboxamido)-3-methylbutyl)phosphonate (**38**). ^{1}H NMR (400 MHz, CDCl$_3$): δ 0.83-0.98 (m, 11H), 1.16-1.19 (m, 3H), 1.56-1.66 (m, 2H), 1.71-1.82 (m, 6H), 1.89-2.03 (m, 5H), 2.60 (s, 3H), 3.75-3.82 (m, 1H), 3.87-4.00 (m, 1H), 4.19-4.27 (m, 1H), 4.51-4.63 (m, 2H), 4.71-4.95 (m, 1H), 7.08-7.19 (m, 6H), 7.24-7.31 (m, 4H), 8.29 (bs, 1H), 8.49 (bs, 1H), 8.96 (bs, 1H). ^{31}P NMR (243 MHz, DMSO-d_6): δ 19.28 (s, 70%), 19.53 (s, 30%). HRMS: calcd for $(C_{34}H_{49}N_4O_6P)H^+$, 641.3462; found, 641.3467. Yield 39%.

diphenyl (1-((S)-1-((S)-3-methyl-2-((S)-2-(methylamino)propanamido)butanoyl)pyrrolidine-2-carboxamido)-3-(methylthio)propyl)phosphonate (**39**). ^{1}H NMR (400 MHz, CDCl$_3$): δ 0.84-0.88 (m, 3H), 1.01 (d, *J* = 6.4 Hz, 3H), 1.21-1.25 (m, 3H), 1.80-2.10 (m, 10H), 2.47-2.54 (m, 2H), 2.60 (s, 3H), 3.69-3.77 (m, 1H), 3.80-3.87 (m, 1H), 4.21-4.28 (m, 1H), 4.51-4.62 (m, 2H), 4.82-4.97 (m, 1H), 7.07-7.20 (m, 6H), 7.24-7.31 (m, 4H), 8.27 (bs, 1H), 8.70-8.80 (m, 2H). ^{31}P NMR (243, MHz, DMSO-d_6): δ 18.41 (s, 60%), 18.74 (s, 40%). HRMS: calcd for $(C_{30}H_{43}N_4O_6PS)H^+$, 619.2714; found, 619.2717. Yield 51%.

diphenyl (1-((S)-1-((S)-2-cyclohexyl-2-((S)-2-(methylamino)propanamido)acetyl)pyrrolidine-2-carboxamido)-3-(methylthio)propyl)phosphonate (**40**). ^{1}H NMR (400 MHz, $CDCl_3$): δ 0.80-1.04 (m, 5H), 1.16-1.22 (m, 3H), 1.54-1.82 (m, 5H), 1.85-2.11 (m, 10H), 2.45-2.55 (m, 2H), 2.59 (s, 3H), 3.68-3.78 (m, 1H), 3.83-3.92 (m, 1H), 4.13-4.22 (m, 1H), 4.49-4.61 (m, 2H), 4.84-5.01 (m, 1H), 7.08-7.20 (m, 6H), 7.24-7.32 (m, 4H), 8.29 (bs, 1H), 8.72-8.85 (m, 2H). ^{31}P NMR (243 MHz, DMSO-d_6): δ 18.44 (s, 66%), 18.71 (s, 34%). HRMS: calcd for $(C_{33}H_{47}N_4O_6PS)H^+$, 659.3027; found, 659.3004. Yield 23%.

diphenyl (1-((S)-1-((S)-3-methyl-2-((S)-2-(methylamino)propanamido)butanoyl)pyrrolidine-2-carboxamido)-2-phenylethyl)phosphonate (**41**). ^{1}H NMR (600 MHz, DMSO-d_6): δ 0.88-0.96 (m, 6H), 1.33 (dd, J = 7.2 Hz, 3H), 1.51-2.06 (m, 5H), 2.51 (s, 3H), 2.93-3.15 (m, 1H), 3.27-3.34 (m 1H), 3.59-3.68 (m, 2H), 3.84-3.90 (m, 1H), 4.38-4.40 (m, 1H), 4.46 (dd, J = 8.4, 4.2 Hz, 1H), 4.81-4.89 (m, 1H), 7.13-7.43 (m, 15H), 8.64-8.72 (m, 1H), 8.81 (bs, 1H), 8.87 (bs, 1H). ^{31}P NMR (243 MHz, DMSO-d_6): δ 18.01 (s, 61%), 18.52 (s, 39%). HRMS: calcd for $(C_{34}H_{43}N_4O_6P)H^+$, 635.2993; found, 635.2991. Yield 50%.

diphenyl (1-((S)-1-((S)-2-cyclohexyl-2-((S)-2-(methylamino)propanamido)acetyl)pyrrolidine-2-carboxamido)-2-phenylethyl)phosphonate (**42**). ^{1}H NMR (600 MHz, DMSO-d_6): δ 0.94-1.20 (m, 5H), 1.32 (d, J = 7.2 Hz, 3H), 1.56-1.98 (m, 10H), 2.51 (s, 3H), 2.91-3.14 (m, 1H), 3.26-3.35 (m, 1H), 3.58-3.69 (m, 2H), 3.82-3.88 (m, 1H), 4.37-4.40 (m, 1H), 4.46 (dd, J = 8.4, 4.2 Hz, 1H), 4.79-4.87 (m, 1H), 7.13-7.42 (m, 15H), 8.63-8.72 (m, 1H), 8.78 (bs, 1H), 8.83 (bs, 1H). ^{31}P NMR (243 MHz, DMSO-d_6): δ 17.94 (s, 65%), 18.47 (s, 35%). HRMS: calcd for $(C_{30}H_{43}N_4O_6P)H^+$, 675.3306; found, 675.3304. Yield 55%.

diphenyl (1-((S)-1-((S)-3-methyl-2-((S)-2-(methylamino)propanamido)butanoyl)pyrrolidine-2-carboxamido)-3-phenylpropyl)phosphonite (**43**). ^{1}H NMR (400 MHz, $CDCl_3$): δ 0.72 (dd, J = 24.4, 6.4 Hz, 3H), 0.98 (dd, J = 27.6, 6.4 Hz, 3H), 1.22-1.24 (m, 3H), 1.82-2.36 (m, 7H), 2.52 (s, 3H), 2.58-2.71 (m, 1H), 2.74-2.81 (m, 1H), 3.66-3.85 (m, 2H), 4.19-4.30 (m, 1H), 4.57-4.65 (m, 2H), 4.75-4.86 (m, 1H), 7.00-7.28 (m, 15H), 8.29 (bs, 1H), 8.71 (bs, 1H), 8.79 (bs, 1H). ^{31}P NMR (243 MHz, DMSO-d_6): δ 18.73 (s, 56%), 18.87 (s, 44%). HRMS: calcd for $(C_{35}H_{45}N_4O_6P)H^+$, 649.3149; found, 649.3153. Yield 45%.

diphenyl (1-((S)-1-((S)-2-cyclohexyl-2-((S)-2-(methylamino)propanamido)acetyl)pyrrolidine-2-carboxamido)-3-phenylpropyl)phosphonate (**44**). ^{1}H NMR (400 MHz, $CDCl_3$): δ 0.81-1.06 (m, 5H), 1.19-1.22 (m, 3H), 1.65-2.28 (m, 12H), 2.56 (s, 3H), 2.60-2.67 (m, 1H), 2.73-2.78 (m, 1H), 3.72-3.82 (m, 1H), 3.88-3.94 (m, 1H), 4.21-4.35 (m, 1H), 4.54-4.63 (m, 2H), 4.65-4.85 (m, 1H), 6.97-7.27 (m, 15H), 8.40 (bs, 1H), 8.71 (bs, 1H), 8.85-8.91 (m, 1H). ^{31}P NMR (243 MHz, DMSO-d_6): δ 18.70 (s, 62%), 18.84 (s, 38%). HRMS: calcd for $(C_{38}H_{49}N_4O_6P)H^+$, 689.3462; found, 689.3453. Yield 62%.

Stability of the free and bound fluorescent probe in fluorescence polarization assay, determination of the Z´ factor.

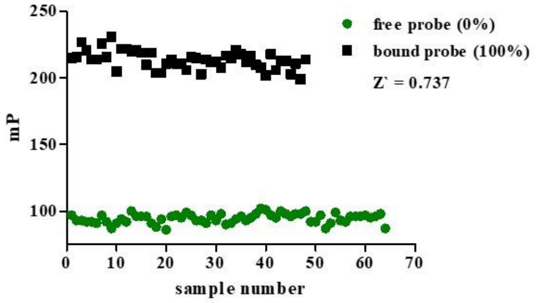

Supplementary Figure S1. Stability of the free and bound fluorescent probe (**4**) used for the fluorescence polarization assay developed for the determination of the potential of obtained phosphoroorganic Smac mimetics to interact with the BIR3 domain of the XIAP protein.

Z´ factor was determined using the equation[1,2]:

$$Z' = 1 - \frac{(3SD_f + 3SD_b)}{(\mu_b - \mu_f)},$$

where SD_f and SD_b are standard deviation of the signal for free (0%) and bound (100%) probe, μ - is the mean value of the signal (mP) obtained for free and bound probe;

[1] Zhang, J. H., Chung, T. D. & Oldenburg, K. R. A Simple Statistical Parameter for Use in Evaluation and Validation of High Throughput Screening Assays. *J Biomol. Screen.* **4**, 67-73; doi:10.1177/108705719900400206 (1999).
[2] Nikolovska-Coleska, Z. et al. Development and optimization of a binding assay for the XIAP BIR3 domain using fluorescence polarization. *Anal. Biochem.* **332**, 261-273; doi:10.1016/j.ab.2004.05.055 (2004).

*The ability of the compound **41** to induce autoubiquitination and proteasomal degradation of cIAP1.*

The dose dependency of compound **41** (0.01, 0.1, 1 and 10 μM) on the degradation of cIAP1 protein after 15 min incubation with MDA-MB-231 cells; **C** – negative control treated only with DMSO.
Western blot analysis of the **41** to induce degradation of cIAP1 protein was performed as described in the Material and Methods section of the manuscript. Shortly, lysates of cells treated with **41** at different concentration ranging from 0.01 to 10 μM were subjected to electrophoresis (25 μg of total protein/lane, reducing conditions) and electrotransfered onto nitrocellulose membrane. After blocking the membrane was incubated with anti-cIAP1 IgG antibodies (1:1,000 in 0.5% skim milk in PBST) and detection anti-rabbit IgG antibodies conjugated with HRP was performed (1:1,000 in 0.5% skim milk in PBST). After visualization of the bands with chemiluminescent substrate, the membrane was washed and incubated with anti-GAPDH mouse IgG antibodies (1:1,000 in 0.5% skim milk in PBST) followed by incubation with anti-mouse IgG HRP labelled detection antibodies (1:5,000 in 0.5% skim milk in PBST) and developed with chemiluminescent substrate (**Supplementary Figure S2**).

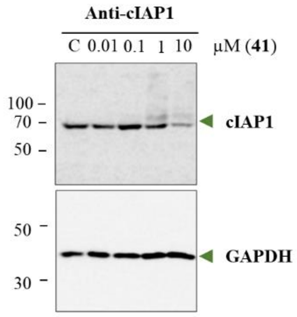

Supplementary Figure S2. The dose response of **41** (0.01, 0.1, 1 and 10 μM) on the degradation of cIAP1 protein after 15 min incubation with MDA-MB-231 cells; **C** – negative control; cells treated with DMSO only.

Synthesis of aryl monoester of α-amino-methylphenyl-phosphonic acid as a phosphoroorganic derivative of N-Me-Ala-Val/Chg-Pro-OH tripeptide.

Synthesis of the aryl monoester of α-amino-methylphenyl-phosphonic acids

Benzyl carbamate (1eq.) and chloro(1,2-phenylenedioxy)phosphite (1eq.) were dissolved in anhydrous toluene followed by a dropwise addition of an phenylacetaldehyde with vigorous stirring. After 10 min the reaction mixture solidified and the reaction was performed under reflux for 5 h. The volatile components were removed under reduced pressure and the products were crystallized from chloroform/MeOH. Cbz-protecting group was removed using 33% HBr/AcOH (2 h, r.t.). The NMR and HRMS analysis revealed the presence of two derivatives: unstable 1,2-phenylenedioxyphosphate derivative (could not be isolated) which spontaneously hydrolyzed into aryl monoesters of α-amino-methylphenyl-phosphonic acid (the main product of the reaction). The synthetic pathway of aryl monoesters of α-amino-methylphenyl-phosphonic acid is shown in **Supplementary Figure S3**.

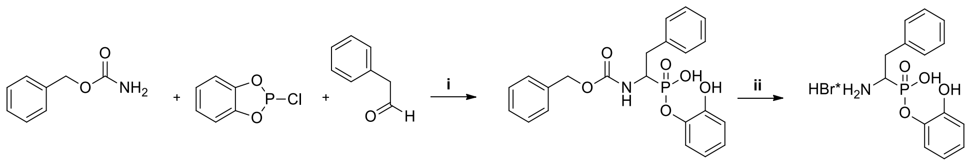

Supplementary Figure S3. Aryl monoester of α-amino-methylphenyl-phosphonic acid synthesis: (**i**) toluene, 5 h, reflux; (**ii**) 33% HBr/AcOH, 2 h, r.t.

Peptide scaffolds (**1** and **2**) were coupled with the α-amino-methylphenyl-phosphine monoester (1.2 eq.) using HBTU as a coupling agent (1.2 eq.) in the presence of DIPEA (5 eq.) in MeCN. The reaction was performed overnight at room temperature. The solvent was removed in vacuum and target compounds (**41m** and **42m**) were isolated directly using HPLC followed by their HRMS analysis (**41m**: calcd for $(C_{28}H_{39}N_4O_7P)H^+$, 575.2629; found 575.2625; **42m**: calcd for $(C_{31}H_{43}N_4O_7P)H^+$, 615.2942; found 615.2946.

*Potency of synthesized phosphoroorganic peptide derivatives **41m** and **42m** to interact with the binding groove of the BIR3 domain.*

Fluorescence polarization inhibition assay and data analysis for **41m** and **42m** compounds was performed as described in the Material and Methods section of the manuscript. Briefly, 50 nM solution of the BIR3 XIAP protein in polarization buffer was added into phosphoroorganic compound solution (from 50 µM to 0.0537 nM). After 15 min incubation at r.t. the fluorescent probe **4** (2 nM) was added. The values were read after 30 minutes from the moment the probe was added. The obtained fluorescence polarization values were used to determine the percentage of inhibition which was plotted as a function of the protein concentration (**Supplementary Figure S4**).

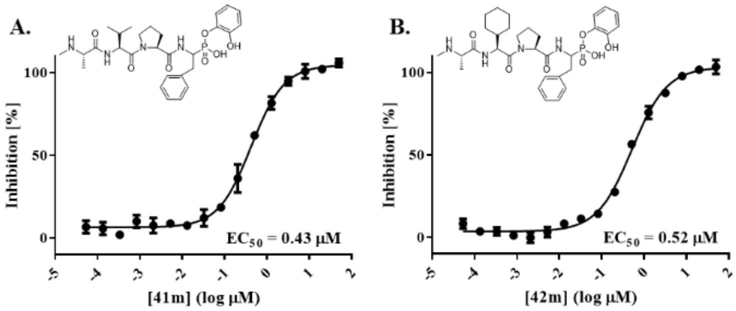

Supplementary Figure S4. Competitive binding curves for aryl monoesters of α-amino-methylphenyl-phosphonic acids.

*Western blot analysis of the potential of **41m** to induce rapid autoubiquitination and proteasome degradation of cIAP1 in MDA-MB-231 breast cancer cells*

Western blot analysis of the **41m** potential to induce degradation of cIAP1 protein was performed as described in Material and Methods section of the manuscript. Shortly, lysates of cells treated with obtained phosphoroorganic compounds or with the reference compounds (10 μM) were subjected to electrophoresis (25 μg of total protein/lane, reducing conditions) and electrotransfered onto a nitrocellulose membrane. After blocking the membrane was incubated with anti-cIAP1 IgG antibodies (1:1,000 in 0.5% skim milk in PBST) and detection anti-rabbit IgG antibodies conjugated with HRP (1:1,000 in 0.5% skim milk in PBST). After visualization of the bands with chemiluminescent substrate, membrane was washed and incubated with anti-GAPDH mouse IgG antibodies (1:1,000 in 0.5% skim milk in PBST) followed by incubation with anti-mouse IgG HRP labelled detection antibodies (1: 5,000 in 0.5% skim milk in PBST) and developed with chemiluminescent substrate (**Supplementary Figure S5**).

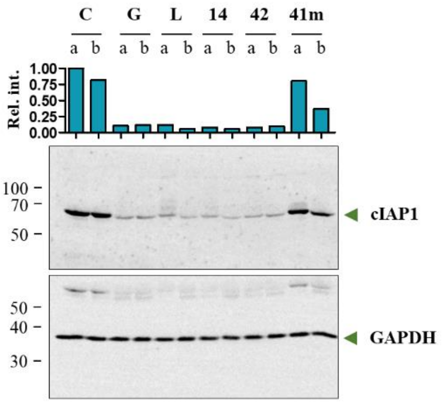

Supplementary Figure S5. Western blot analysis of MDA-MB-231 cell lysates subjected to incubation with reference compounds: **G** – GDC-0152, **L** – LCL-161, selected phosphoroorganic peptide derivatives (**14**, **42** and **41m**) and a negative control treated only with DMSO (**C**); a and b – samples taken after 15 and 120 min of incubation respectively. Relative intensity was calculated assuming the intensity of the band corresponding to control (**C**) equal to 1 while the membrane background equal 0.
